# Effects of Baobab fruit powder on gut and cardiometabolic health in obesity—Protocol for a randomised placebo-controlled trial

**DOI:** 10.1371/journal.pone.0328774

**Published:** 2025-08-13

**Authors:** Sylvia Riedel, Keren de Buys, Abegail M. Tshivhase, Amy E. Mendham, Pieter Venter, Fatima Hoosen, Lara R. Dugas, Caroline D’Alton, Jody A. Rusch, Bianca Southon, Carmen P. Pheiffer, Rabia Johnson, Tarylee Reddy, Nonhlanhla Yende-Zuma, Christo J. F. Muller, Joel A. Dave, Julia H. Goedecke

**Affiliations:** 1 Biomedical Research and Innovation Platform, South African Medical Research Council, Tygerberg, South Africa; 2 Centre for Cardio-metabolic Research in Africa, Division of Medical Physiology, Faculty of Medicine and Health Sciences, Stellenbosch University, Cape Town, South Africa; 3 Riverland Academy of Clinical Excellence, Riverland Mallee Coorong Local Health Network, South Australia Health, Australia; 4 Health through Physical Activity, Lifestyle and Sport Research Centre, Division of Physiological Sciences, Department of Human Biology, Faculty of Health Sciences, University of Cape Town, Cape Town, South Africa; 5 Public Health Sciences, Loyola University Chicago, Maywood, Illinois, United States of America; 6 Division of Epidemiology and Biostatistics, School of Public Health, University of Cape Town, Cape Town, South Africa; 7 Division of Physiological Sciences/Division of Sports Medicine, Department of Human Biology/Department of Family, Community and Emergency Care, University of Cape Town, Cape Town, South Africa; 8 Division of Chemical Pathology, Department of Pathology, University of Cape Town, Cape Town, South Africa; 9 C17 Laboratory, National Health Laboratory Service, Groote Schuur Hospital, Cape Town, South Africa; 10 Department of Obstetrics and Gynaecology, Faculty of Health Sciences, University of Pretoria, Pretoria, South Africa; 11 Biostatistics Research Unit, South African Medical Research Council, Durban, South Africa; 12 Department of Biochemistry and Microbiology, University of Zululand, KwaDlangezwa, South Africa; 13 Division of Endocrinology, Department of Medicine, University of Cape Town, Groote Schuur Hospital, Cape Town, South Africa; Texas A&M University, UNITED STATES OF AMERICA

## Abstract

Despite its commercial availability, its high fibre, vitamin C and polyphenol content, there are limited scientific studies exploring the cardiometabolic effects of Baobab fruit powder (BFP) in humans. Due to its high fibre content, BFP may offer a potential intervention to reduce intestinal barrier dysfunction and therefore mitigate cardiometabolic risk. A randomized, double-blind, placebo-controlled trial will be conducted with 50 apparently healthy participants living with obesity. Participants will consume either 16 g of BFP or an isocaloric placebo daily for 45 days. The primary outcome will be intestinal permeability determined using the urinary lactulose/mannitol test. Secondary outcomes include blood biomarkers in intestinal permeability (lipopolysaccharide (LPS), intestinal fatty acid binding protein (IFABP), soluble cluster of differentiation 14 (sCD14) and LPS-binding protein (LBP)), microbiota diversity and composition and cardiometabolic risk markers including glucose levels, blood lipid profiles and blood pressure. Liver and kidney function will be monitored at baseline, after 2 weeks and following 45 days of consumption as safety outcomes. The study protocol ensures rigorous, weekly monitoring of participant compliance and tolerability, along with careful tracking of potential adverse events. Intention-to-treat analysis and mixed effects models will be employed for statistical analyses. Potential selection bias and participant dropout are addressed through thorough recruitment strategies and predefined sample size calculations. This research will contribute to the growing body of knowledge on dietary interventions in the context of cardiometabolic risk, particularly in populations at risk for developing metabolic disease.

South African Clinical Trial Registry - SANCTR, DOH-27-062024-8061; Pan African Clinical Trial Registry – PACTR202308727853680

## Introduction

Baobab (*Adansonia digitata* L.) is an iconic tree indigenous to sub-Saharan Africa. Baobab leaves, seeds, bark, roots and fruit pulp are traditionally used and/or consumed due to their nutritional value and anecdotal health benefits [1,2]. In 2008, Baobab fruit powder (BFP), derived from the fruit of the baobab tree, was authorised as a novel food ingredient in the European Union (EU) and has since been commercially available worldwide. The powder typically contains significant levels of soluble fibre, polyphenols, vitamin C, citric acid and minerals such as calcium, magnesium, potassium and iron [1,3–9]. However, a limited number of studies have explored potential effects of BFP in humans.

To our knowledge few studies have reported potential pre-biotic effects of BFP, showing increased production of short chain fatty acids (SCFA), such as acetate, propionate and butyrate, in faecal cultures from three human donors *in vitro* [9]. Increased intestinal permeability is now a well-established factor implicated in the pathophysiology of metabolic diseases such as obesity and type 2 diabetes, as well as cardiovascular disease [10–14]. The underlying mechanism linking increased intestinal permeability with metabolic diseases is likely the translocation of sub-clinical levels of gut-derived bacterial products such as lipopolysaccharide (LPS) from the intestinal lumen into the blood stream, activating immune responses and resulting in low-grade inflammation characterised by increased levels of C-reactive protein and cytokines such as tumour necrosis factor alpha (TNFα) and interleukin-6 (IL-6) [10,12]. Importantly, interventions such as drugs or herbal formulations with intestinal barrier-protective properties have been shown to improve intestinal permeability [15,16].

Intricately linked with intestinal permeability and homeostasis are commensal microbiota composition and diversity, which are also decreased and/or altered in obesity and type 2 diabetes [17–20]. Diet, and specifically dietary fibre, profoundly affect microbiota composition through selection of saccharolytic bacterial strains that utilise and digest fibres [21,22] and several studies have shown that dietary fibre supplementation can affect microbial composition as well as intestinal barrier function in humans [23–26].

Improved intestinal permeability and microbial composition may modulate dysglycaemia and possibly weight gain. Studies involving healthy adults showed promising acute effects of Baobab fruit extracts on lowering postprandial glycaemia [27,28], while Coe & Ryan [29] reported a reduced 3 hour postprandial insulin response and Garvey *et al.* [30] reported decreased hunger over the 2 hours post consumption.

Based on the reports on the pre-biotic properties in conjunction with the high fibre and polyphenolic content of BFP, we hypothesise that BFP will decrease intestinal permeability, increase intestinal microbial diversity and subsequently lead to improvements in cardiometabolic risk factors. Accordingly, the aim of this study is to perform a randomised double-blind placebo-controlled trial to examine the effects of consumption of BFP on intestinal permeability in participants living with obesity. As secondary aim we will assess the effects of BFP on gut microbiota and cardiometabolic risk factors, as well as confirm the safety for consumption of BFP in humans.

The objectives of this study are to measure the following changes in response to the 45-day BFP intervention compared to the control group:

- changes in intestinal permeability using the urinary lactulose/mannitol (Lac/Man) ratio (primary outcome)
- changes in blood biomarkers of intestinal permeability (secondary outcomes)
- changes in the composition and diversity of intestinal microbiota profiles using 16S ribosomal RNA gene sequencing (secondary outcomes)
- changes in cardiometabolic risk markers including anthropometric measurements (such as body mass index (BMI), waist and hip circumference), blood lipid profiles, fasting glycaemia, insulin resistance, inflammation and blood pressure (secondary outcomes)
- changes in liver (aspartate transaminase (AST), alanine transaminase (ALT), alkaline phosphatase (ALP), gamma glutamyl transpeptidase (GGT)) and kidney (creatinine) function and gastrointestinal symptoms (safety outcomes)

## Methodology

### Study design

In this randomised double-blind, placebo-controlled trial, 50 apparently healthy women and men living with obesity will be randomised at a 1:1 ratio into an experimental (BFP) or a control (placebo) group. Both BFP and the isocaloric placebo will be consumed daily for 45 days.

**Fig 1:**
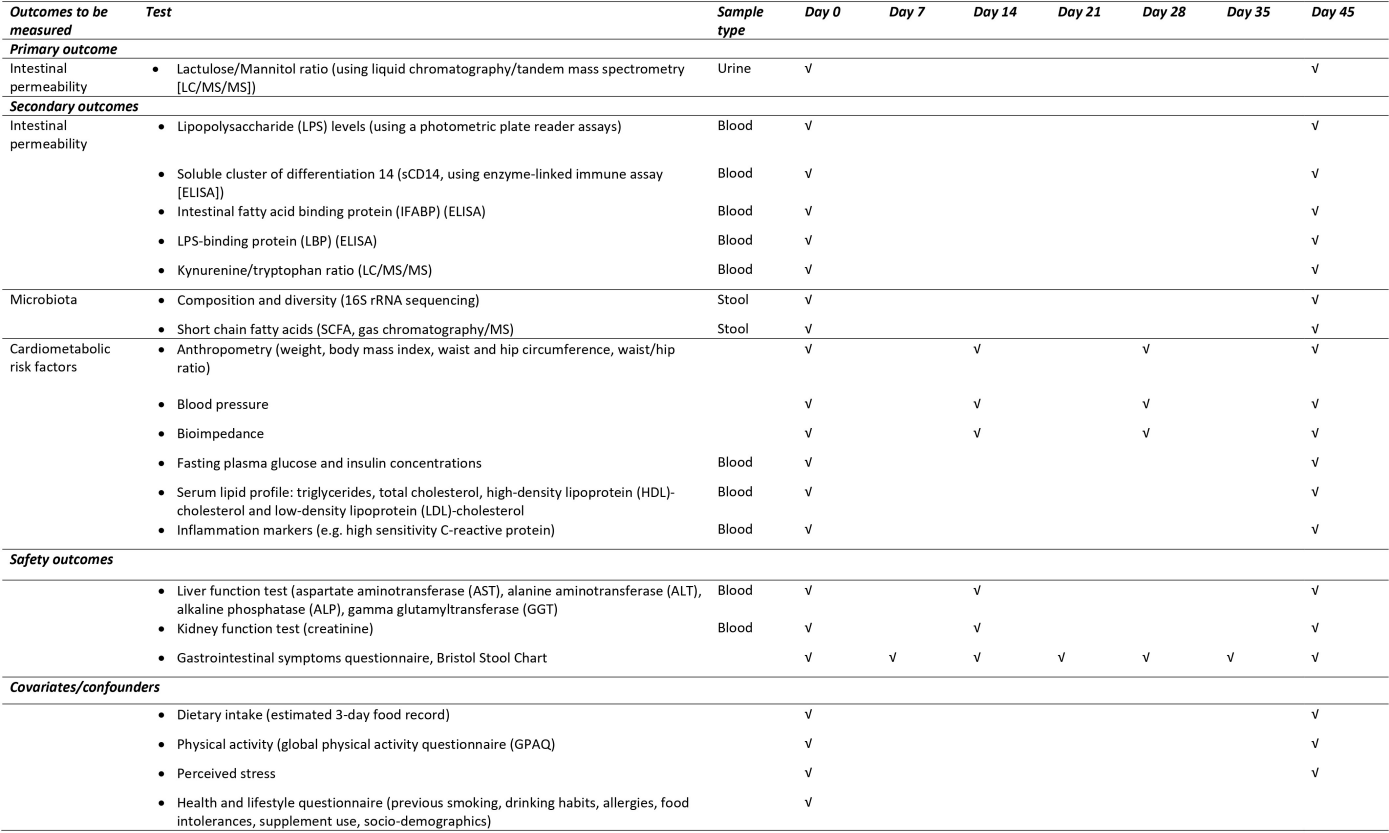
Study schedule of enrolment, interventions and assessment for the proposed randomised placebo-controlled trial.

**Fig 2:**
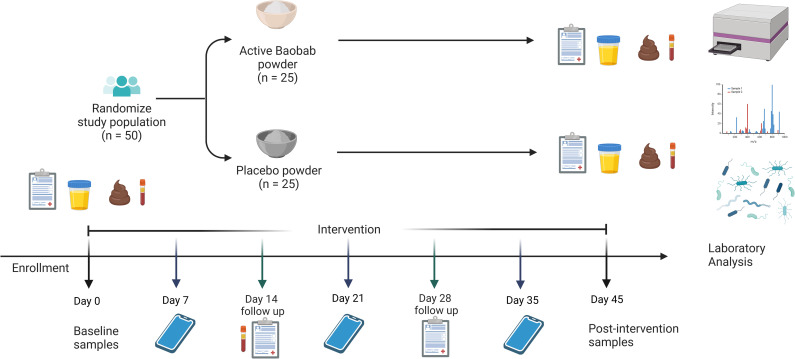
Study outline for the proposed randomised placebo-controlled trial. Participants meeting the inclusion criteria will be invited to attend the baseline testing session where a urinary intestinal permeability test (lactulose/mannitol test) will be conducted. Socio-demographic, health, physical activity, and dietary data will be entered directly into the electronic data capturing tool (REDCap). Anthropometric (Body mass index, waist and hip circumference) and blood pressure measurements will also be entered into REDCap. Fasting blood samples will be taken to determine safety outcomes (liver and kidney function), as well as blood biomarkers of intestinal permeability and inflammation, fasting glycaemia, insulin resistance and lipid profiles. A stool sample will be requested for analysis of microbiota composition and diversity. The Baobab fruit powder (BFP) group will consume 16 g of BFP and the control group will consume 13.5 g placebo (tapioca flour and citric acid) with similar taste and caloric content daily for 45 days. The testing procedures will be repeated at the 45-day follow-up visit. Participant compliance, tolerability, acceptability and symptoms will be monitored weekly through alternating telephonic contact and visits to the research facility. (Fig 2 was created with BioRender.com).

The schedule of enrolment, intervention and assessments is outlined in Fig 1. Prior to and following the 45-day intervention, participants will complete one testing session, during which the primary and secondary outcomes as well as potential confounders will be measured. To assess safety of BFP, liver (liver enzymes) and kidney (estimated glomerular filtration rate, GFR) function will be assessed at baseline, 2-weeks into the intervention and following the 45-day intervention. Gastrointestinal symptoms will be assessed weekly using validated questionnaires. Participants will be monitored weekly by alternating telephonic contact and visits to the research facility to confirm compliance, acceptability and tolerability. Adverse events, such as allergic reactions, and incidence of other potentially confounding health issues such as illnesses that require extended use of medication will also be assessed. The participants will be encouraged to maintain their usual dietary and lifestyle behaviours during the intervention period. Participants will be reimbursed for their time and travel costs. The overview of the study design is described in Fig 2.

Ethical approval was obtained from the Human Research Ethics Committees (HREC) at the South African Medical Research Council (reference EC024–11/2022) and University of Cape Town (287/2023). The study will be performed in accordance with the principles of South African Good Clinical Practice (SA GCP 2020 version 3), the Declaration of Helsinki (1964, as amended in Fortaleza Brazil, 2013), the ICH Good Clinical Practice (GCP) and the applicable laws of South Africa. Participants will be required to provide written informed consent prior to participation in the screening and the research study. The trial has been registered with the South African Clinical Trial Registry (SANCTR, DOH-27-062024-8061), the Pan African Clinical Trial Registry (PACTR202308727853680) and received approval from the South African Health Products Regulatory Authority (SAHPRA, Ref 20231121).

#### Participant recruitment and screening.

Participant recruitment and testing sessions will be conducted at the research facility at the University of Cape Town (UCT) in Newlands, Cape Town, South Africa. Awareness will be raised through sharing advertisements via mailing lists and using social media posts on Facebook, LinkedIn and Twitter. Flyers will be distributed at UCT and in the surrounding community to attract participants who are living close to the research facility. Prior to recruitment into the study, participants will undergo screening to confirm their eligibility.

Participants will be invited to attend a screening visit where the purpose of the study and the procedures will be explained in detail and queries regarding the study will be answered. Participants will then sign the informed consent form if they agree to participate in the study and complete a detailed screening questionnaire listing compliance with inclusion (age between 25 and 55 years, body mass index (BMI) ≥ 30 kg/m^2^) and exclusion criteria as detailed in Table 1. Participants with gastrointestinal diseases, previous gastrointestinal surgery, chronic diseases, those taking chronic medication, smokers, pregnant or breastfeeding women will be excluded (Table 1). Body weight, in lightweight clothing without shoes will be measured to the nearest 0.1 kg using a digital scale (Tanita TBF-410GS Total Body Composition Analyzer, Tokyo, Japan), height will be measured to the nearest 0.1 cm using a wall-mounted stadiometer (Holtain, Ltd., Crosswell, UK) [31,32] and BMI will be calculated as weight (kg)/height (m)^2^. After the participant had been seated for at least five minutes, three blood pressure readings will be taken at two-minute intervals using a digital blood pressure monitor (Rossmax, Heerbrugg, Switzerland). After an overnight (10–12 hours) fast, blood samples will be drawn for the measurement of screening parameters including fasting plasma glucose and serum lipid profile (high density lipoprotein (HDL)-cholesterol, low density lipoprotein (LDL)-cholesterol, total cholesterol and triglycerides). Participants that meet all inclusion and exclusion criteria will be enrolled in the study.

**Table 1:** Inclusion and exclusion criteria.

	*How is it determined:*	*Motivation:*
***Inclusion criteria***		
Ability to sign informed consent	Confirmation through questions that participants understand what will be asked of them	Ethical requirement.
Age between 25 and 55 years	Date of birth	This younger age group may be at risk but less likely to display co-morbidities or non-communicable diseases associated with obesity, which could be considered confounders.
Body Mass Index (BMI) ≥ 30 kg/m^2^	Anthropometric measurements	BMI above 30 kg/m^2^ has been associated with increased risk for increased intestinal permeability [33,34].
Weight stable (less than 5 kg weight change in the last 6 months)	Self-reported change in clothes size	Weight change affects cardiometabolic risk factors.
***Exclusion criteria:***		
History of gastrointestinal diseases (e.g., irritable bowel syndrome, coeliac disease, Crohn’s disease, Ulcerative Colitis) and gastrointestinal surgery	Self-reported	Effect on intestinal permeability and gut microbiota.
Current smoker (including e-cigarettes) or user of chewing tobacco	Self-reported	Confounding factor for cardiometabolic risk.
Current acute bacterial or viral infection	Self-reported	Potential effect on intestinal permeability and gut microbiota.
Pregnancy or breastfeeding	Self-reported	Ethical considerations
Regular use of chronic (defined as either daily or regular intake at least 3 times per week) medication:		Potential effects on intestinal permeability and gut microbiota.
prescription medication for obesity, chronic non-communicable and infectious diseases	Self reported	
immunosuppressants	Self reported	
frequent use of over-the-counter drugs such as non-steroidal anti-inflammatory drugs and laxatives	Self reported	
Known chronic diseases (with or without treatment with prescription medication) or screen-detected diseases, e.g.,:		Potential effects on intestinal permeability and gut microbiota. Chronic diseases could provide significant confounding factors and may therefore reduce the statistical power of the study.
Autoimmune diseases	Self reported	
Hypertension (BP > 140/90 mm Hg)	Self-reported, blood pressure will be measured on site	
Type 1 or Type 2 diabetes (FBG > 7 mmol/l)	Self-reported, FBG will be assessed	
Dyslipidaemia (TG > 3.0 mmol/l, LDL > 4.0 mmol/l; Participants with TG > 1.7 mmol/l or LDL > 3.0 mmol/l and Framingham risk scores of ≥ 3 for men and ≥ 5 for women [moderate risk and above] [35])	Self-reported, serum lipids will be measured and risk assessed according to Framingham risk scores	
Heart disease	Self reported	
Stroke	Self reported	
Reproductive diseases (e.g., endometriosis or polycystic ovary syndrome)	Self reported	
Thyroid diseases	Self reported	
Infectious diseases (such as HIV)	Self reported	Potential effects on intestinal permeability and gut microbiota
Severe allergies, known allergy to baobab [36] or tapioca flour, and food intolerances (e.g., intolerance to sugar alcohols [mannitol] or lactose intolerance)	Self reported	To avoid risk of sensitisation and new allergic reactions; Food intolerances affect intestinal permeability and can be confounding factors.
Oral antibiotic use, fibre, pre- and/or probiotic supplement use in the preceding 3 months [37,38]	Self reported	Effect on intestinal permeability and gut microbiota.

Abbreviations: BP – blood pressure; FBG – fasting blood glucose; HIV – human immunodeficiency virus; LDL – low density lipoprotein; TG – triglycerides.

Participant recruitment commenced in July 2024. As of June 2025, recruitment, enrolment, data collection and follow up for this trial are ongoing with recruitment expected to be completed by September 2025 and follow up and data collection completed by October 2025.

#### Intervention and randomisation.

Baobab fruit powder will be supplied by the African Baobab Alliance (ABA), a not-for profit sector organisation representing multiple stakeholders in the Baobab sector, including Baobab fruit harvesters from underserved rural Southern African communities. The BFP provided for this study is a certified organic and natural product and is available in South African supermarkets. It is derived from fruit of the baobab tree that are free-harvested by rural harvesters in the Limpopo Province, South Africa. The BFP surrounds the seeds that are protected by a hard shell. The processing of the fruits includes opening the fruit, separating the seeds from the powder and then milling, sieving and packaging the powder for consumption.

For the trial, the BFP group will consume 16 g BFP daily for 45 days, while the control group will consume 13.5 g placebo containing tapioca flour and citric acid with a similar appearance and taste to the BFP. The 16 g dose of BFP was selected based on serving suggestions of commercially available products which vary between 8 and 24 g per day. The selected 16 g dose is equivalent to 2 heaped tablespoons and will deliver approximately ~8 g of dietary fibre per day. Previous studies reported long-term use (tolerability) [39], palatability [30], significant changes in postprandial glycaemia [27,28] and insulin response [29] with dosages of 16 g and above. The duration (45 days) was based on studies reporting statistically significant differences in the Lac/Man ratio between the respective intervention and placebo-treated groups [15,40]. To our knowledge, there were no similar studies using a placebo-controlled design to guide the choice of the placebo treatment. The texture and appearance of tapioca flour is similar to BFP, which is important when considering that some participants may be familiar with BFP. Furthermore, tapioca flour does not contain significant levels of dietary fibre (~0.6 g per 100 g), consists predominantly of carbohydrates and contains minimal amounts of other nutrients (https://www.natureschoice.co.za/shop/tapioca-flour-2/). As our study hypothesis is based on the fibre content of BFP (~50%), tapioca flour can be regarded as inert in this context. Therefore, the placebo was chosen to contain tapioca flour (12 g) to mimic the appearance and citric acid (1.5 g) was chosen to mimic the taste of BFP. Citric acid is an organic acid commonly found in fruits and specifically also in Baobab fruit powder [1,6]. The dose for the placebo (13.5 g per day containing ~0.07 g fibre) was calculated to deliver the same energy content as the 16 g BFP (~160 kJ), which is equivalent to half a slice of bread and thus contributes a minor addition to the daily energy intake of our participants. The carbohydrates in tapioca flour are unlikely to have a significant glycaemic impact, however, fasting glycaemia and insulin resistance will be assessed in all participants before and after the intervention. The sachet contents of BFP and placebo can be suspended in up to 1 L of water or added to other cold beverages such as smoothies and juices, or even to cereals. The participants will be encouraged to maintain their usual dietary and lifestyle behaviours during the intervention period. While our study hypothesis is based on the fibre content, BFP, as a food, is also a source of minerals and vitamins [1,6], which may potentially affect gut and cardiometabolic health markers. These vitamins and minerals are integral to the fruit pulp and its properties and should not be considered as confounders. The daily dose of 16 g BFP to be consumed during the intervention would deliver ~20 mg vitamin C, ~ 290 mg Potassium, ~ 30 mg Magnesium and ~50 mg Calcium, based on typical nutritional analysis data [6].

The intervention and placebo powder were packaged in foil sachets protected from light by Joypak, Cape Town, South Africa, with non-identifiable labels to ensure that both study staff and participants will be blinded to the group randomisation/allocation. Participants will be block randomised at a 1:1 ratio with random permuted block sizes of 2 and 4 while applying *a priori* stratification for sex. Researchers will use the randomisation module on RedCap to allocate a randomisation number to the participants on the day of baseline testing. Participants, investigators, analysts and statistician will be blinded to the group allocation. In the event that unblinding becomes necessary, two independent SAMRC staff members who are not directly involved in the study will be able to identify specific codes.

#### Pre- and post-intervention testing.

On the baseline and follow-up testing days, participants will attend the research facility following a 10–12 hour overnight fast. For 48 hours prior to these testing sessions, participants will be requested to avoid strenuous exercise, alcohol, non-nutritive sweeteners (e.g., in chewing gum and beverages), spicy food, caffeine (including tea), sporadic over-the-counter medication (such as headache pills or other nonsteroidal anti-inflammatory drugs) and supplement use [37,38].

**Stool sampling:** Participants will be given an EasySampler kit to take home for the collection of a pre- and post-intervention stool sample for microbiota analysis. The fresh sample will be aliquoted at the research facility within 6 hours. A pea-sized aliquot of the fresh sample will be removed using a sterile swab and stored in a cryovial prior to microbiota analysis. Approximately 1 g of stool will be weighed for SCFA analysis and all samples will be stored at −80°C.

**Blood sampling:** A fasting venous blood sample (~45 ml) will be collected for the subsequent determination of plasma glucose, serum insulin, serum lipids (triglycerides, total cholesterol, HDL cholesterol and LDL cholesterol), inflammatory markers (e.g., high-sensitivity C-reactive protein [hs-CRP] and biomarkers of intestinal permeability (e.g., LPS, LPS-binding protein [LBP], soluble cluster of differentiation 14 [sCD14], intestinal fatty acid binding protein [IFABP]) as well as safety endpoints (creatinine, aspartate aminotransferase [AST], alanine aminotransferase [ALT], alkaline phosphatase [ALP], gamma glutamyltransferase [GGT]).

**Lactulose/Mannitol test:** The intestinal permeability lactulose/mannitol (Lac/Man) test will be conducted as described by Seethaler *et al.* [37] and Khoshbin *et al.* [38]. A urine sample (30 ml) will be collected prior to the test to account for baseline levels of mannitol and lactulose. Lactulose (5 g, Dulphalac, Clicks, South Africa) and mannitol (2 g, Merck Life Science, Darmstadt, Germany) will be dissolved in 200 ml water to be consumed within 5 minutes by the participants to initiate the test procedure. Cumulative urine will be collected for 24 hours, of which the first 5 hours will be collected at the research facility (estimated small intestinal permeability). Participants will be provided with water and a meal after 2 hours. The participants will collect their urine for a further 19 hours in provided containers (estimated large intestinal permeability). The 5-hour and 24-hour urine volumes will be recorded, and aliquots will be stored at −80 °C before liquid chromatography/mass spectrometry (LC/MS/MS) for the quantification of lactulose and mannitol.

**Blood pressure and anthropometry:** Determination of blood pressure, weight and height measurements are described in the screening section. Waist circumference will be measured in the mid–axillary line at the midpoint between the lower margin of the last palpable rib and the top of the iliac crest at the end of normal exhalation, and hip circumference at the largest protrusion of the buttocks. Both will be measured to the nearest 0.1 cm. Whole body composition will be estimated using a Quantum Legacy Bioelectrical Impedance Analyser (BIA, RJL Systems, USA). Blood pressure, weight, hip and waist circumference and whole-body composition will be taken every 2 weeks at the participant visits to the research facility.

**Lifestyle and sociodemographic characteristics:** Lifestyle, health and socio-demographic information will be collected using validated questionnaires [31,32]. This includes age, sex, self-reported ethnicity and family history of disease. Gastrointestinal symptoms [41,42] and stool consistency will be assessed (Bristol stool chart) [43]. Perceived stress [44] and lifestyle factors, including previous smoking habits and alcohol intake will also be determined. Socio-economic status will be assessed based on factors such as education level, occupation, employment status and housing. In addition, physical activity will be estimated using the validated Global Physical Activity Questionnaire (GPAQ) [45]. Dietary intake will be estimated using a 3-day food record, including 2 non-consecutive weekdays and one weekend day. Nutrient intake will be analysed using the South African Food Composition Database System (SAFOOD, the South African Food Composition Database, South African Medical Research Council, Cape Town, South Africa) [32].

#### Safety and progress monitoring.

Participant compliance, tolerability, acceptability and symptoms will be monitored weekly through alternating telephonic contact and visits at the research facility. Participants will be asked about any side effects, any adverse events, including gastrointestinal symptoms, and stool consistency. Should any adverse events occur, the participant needs to contact study staff and will be referred to the study physician and to a healthcare facility, if necessary. During the biweekly in-person visits, participants will be requested to return used and unused sachets for accountability and receive the next batch of investigational product. In the event that a participant develops an unrelated illness (such as a cold of flu) and requires medication, the study physician will assess whether the participant needs to be excluded.

At the first in-person follow up session (after 2 weeks), a blood sample for safety endpoints, including kidney (creatinine) and liver function (AST, ALT, ALP, GGT), will be collected.

A steering committee consist of four independent and experienced scientists and two investigators who are part of the trial team. This committee will provide oversight over the conduct of the trial and to safeguard the interests of study participants. The steering committee will be responsible for (i) assessing the safety aspects of study procedures (e.g., review of the research protocol, informed consent documents and plans for data safety and monitoring), (ii) for monitoring the overall conduct of the study (periodic assessment of participant recruitment and retention) and (iii) report on safety and progress of the trial. Should any participants present with abnormal clinical values before or during the study, they will be referred to the study clinician and assessed for further referral and/or treatment.

The stopping rules for this study include the following:

Should there be 10 or more serious adverse events (SAEs) that are judged by the study clinician to be related to the investigational product, the study will be stopped. Participation in the trial will be stopped for individual participants should there be any renal dysfunction detected, any study-related allergic reaction, liver function test results more than 2-fold above the upper normal limit or when participants present with gastrointestinal symptoms with no other obvious cause that warrant discontinuation of the investigational product and further participation in the study. These gastrointestinal symptoms include abdominal pain, nausea or diarrhoea more than 3 times per week in 2 consecutive weeks and significantly more frequent than at baseline.

### Biochemical analyses

#### Urinary Lactulose/Mannitol ratio.

Ultra-high performance liquid chromatography (UHPLC, Shimadzu LC-40D, Kyoto, Japan) coupled to a triple quadrupole mass spectrometer (MS/MS; AB Sciex Qtrap 6500+) will be used to determine the urinary Lac/Man ratio to evaluate the intestinal barrier function as described by Gervasoni *et al.* [46]. The UHPLC separation will be performed using an ACQUITY UPLC BEH amide column (Waters, Milford, MA, USA) and sample preparation will be limited to a “dilute and shoot” method using labelled internal standards (D-Mannitol-1-^13^C,1–1-d_2_ and Lactulose-^13^C_12_, Merck Life Sciences, Darmstadt, Germany) which will provide the highest reliability in terms of reproducibility and accuracy. Due to the low ionization efficiency of these sugars, a comparison between electrospray ionization (ESI) and chemical ionization (APCI) will be conducted.

#### Blood analyses.

Plasma glucose and serum insulin, lipids, hs-CRP and safety markers (creatinine, ALP, AST, ALT, GGT) will be analysed at an ISO 15189−2012 accredited clinical laboratory (National Health Laboratory Services, Groote Schuur, Cape Town) according to standard procedures using a Roche Cobas® e501 analyser (Roche Diagnostics, Basel, Switzerland). Briefly, plasma glucose will be determined using colorimetric assay (Randox, Gauteng, South Africa) and creatinine, ALP, AST ALT and GGT will be measured using colorimetric enzyme assays (Roche Diagnostics, Basel Switzerland). Serum insulin, lipid profile and hs-CRP will be measured using immunochemiluminometric assays (IMMULITE 1000 immunoassay system, Siemens Healthcare, Midrand, South Africa). LDL-cholesterol concentrations will be calculated using the Friedewald equation [47]. The HOMA-IR index will be calculated using fasting plasma glucose and insulin levels according to Matthews *et al.* [48]. Remaining SST and EDTA tubes will be centrifuged, and serum and plasma aliquots will be stored at −80 °C until further analyses and for long-term storage.

Biomarkers of intestinal permeability will be measured in plasma or serum samples in duplicate using commercially available enzyme linked immunosorbent (ELISA) kits. The following targets were chosen for intestinal permeability and will be measured at baseline and following the 45-day intervention:

- plasma LPS levels will be assessed using a commercial kit (Thermo Fisher Scientific, Waltham, MA, USA) and a plate reader (Spectramax i3x, Molecular Devices, San Jose, CA, USA).
- serum LBP, sCD14 and IFABP will be measured using commercially available Sandwich ELISA assay kits (R&D Systems, Minneapolis, MN, USA) using a plate reader (Spectramax i3x, Molecular Devices, San Jose, CA, USA).
- the kynurenine/tryptophan ratio, which is an indicator of indoleamine 2,3-dioxygenase (IDO) activity, will be quantified using the LC/MS/MS method described by Fuertig *et al.* [49] in plasma samples.

#### Faecal short chain fatty acids.

Short chain fatty acids in stool samples will be determined as described by Zhu *et al*. [50]. Stored stool samples will be thawed, acidified, and homogenized with ceramic beads. After centrifugation, the supernatant will be acidified, spiked with an internal standard, and extracted using diethyl ether. The organic phase will be transferred to a tube with sodium sulphate, vortexed, centrifuged, and dried. The dried extract will then be reconstituted with diethyl ether before being analysed via GC/MS to quantify short-chain fatty acids (SCFAs) such as butyric, propionic, acetic, and valeric acids, both individually and as total SCFAs. The quantification will be performed using calibration curves generated from known standards, allowing for precise measurement in µg/g. The GC/MS system will identify compounds based on retention times and fragmentation patterns, with parameters like ionization voltage and temperature optimized for enhanced sensitivity. An Agilent DB-FATWAX UI column (30m, 0.25 mm, 0.25µm) will be used on a Perkin Elmer Clarus 690 GC, employing electron ionization for mass spectrometry analysis.

#### Faecal microbiota analysis.

Nucleic acids will be extracted using the MagMax Microbiome Ultra kit (Thermo Fisher Scientific, Waltham, MA, USA) on the Kingfisher robotic platform. PCR of the 16S rRNA V4-5 region will be performed and sequenced using 2 x 150 paired-end multiplexed sequencing on the NovaSeq 6000. The DNA extraction and 16S ribosomal RNA metagenomic sequencing will be conducted by Dr Jack Gilbert, Director of the Microbiome and Metagenomics Center, at the University of California, San Diego, USA. All amplicon sequencing data will be analysed using the QIIME2 platform [51]. For these data, all sequence data will be quality filtered and de-multiplexed, followed by DeBlur amplicon sequence variant (ASV) calling to obtain unique sequences, which will be compared against various databases to allow for annotation.

### Storage of samples for future use

Permission to store blood, stool and urine samples at −80 °C for future biomedical analyses, specifically exploring diseases such as gastrointestinal diseases, type 2 diabetes, cardiovascular disease, will be obtained from the participants in separate written informed consent forms. Approval from the Human Research Ethics Committee at the SAMRC and University of Cape Town will be obtained before any additional research is performed on stored blood, stool and urine samples.

#### Data management.

Confidentiality will be strictly maintained. Participant names will be removed from all data, and each participant will receive a unique participant identification number to be used for sample and data analysis on the electronic data management system. Data will be entered directly into the electronic data capturing tool Research Electronic Data Capture (REDCap) [52,53] on secure IT infrastructure with backup features at the SAMRC. Personal data is stored separately from the research data on RedCap. Study team members will receive password-protected permission to access the database as required. The PI will have access to personal data to ensure that individual test results can be communicated to the participants at the end of the trial. A data manager will be responsible for ensuring quality and completeness of the data collected. Data and samples will be stored for 10 years and will be made available upon request.

### Statistical analysis

#### Sample size determination.

The study is powered on the primary study endpoint, the Lac/Man ratio, which is based on the data reported by del Piano *et al.* [40] for a similar treatment period. Considering a mean ± standard deviation (SD) difference in the Lac/Man ratio between treatment and control groups (0.015 ± 0.006 versus 0.021 ± 0.007) after the 45-day treatment [40], significance level of p < 0.05 (two-sided test) and power of 80%, 20 participants per group would be sufficient for our study to detect significant differences. When factoring in a drop-out rate of 25% (5/25), 25 participants were selected per group. In the unlikely event that there is a dropout of greater than 25%, we will recruit additional participants until we meet the required sample size.

#### Statistical analysis.

Results will be presented as means ± SD or medians and interquartile ranges for normally and skewed data, respectively. Non-normally distributed data will be transformed for parametric analysis. Intention to treat analysis approach will be used. A mixed effects model will be used to compare within and between groups (control and BFP) differences in primary and secondary outcome variables adjusting for potential confounders as outlined in Table 1. Data will be analysed using STATA SE (version 17, StatCorp, Texas, USA).

Statistical comparisons for taxonomic differences will be performed using Songbird [54], an experimentally validated, compositionally coherent differential abundance method. Statistical visualization of reference frames from Songbird will be performed using Qurro [55], which forms part of a compendium of PCA/PCoA visualization tools such as EMPress [56] and EMPeror [57], designed for the analysis of microbiome datasets. For longitudinal data we will employ volatility analyses, such as semivariograms [58] and Compositional Tensor Factorization [59], to decompose time-series responses into components acting on different timescales. Using these approaches, we will identify taxa that are associated with Baobab fruit powder supplementation, faecal SCFA production, biomarkers of intestinal permeability (LPS, LBP, sCD14, IFABP) and cardiometabolic parameters including glucose, insulin, lipid profiles, inflammatory markers across intervention groups.

## Discussion

Although BFP has been consumed by humans for centuries [60], to the best of our knowledge, this is the first study to explore the potential effects of regular consumption of BFP on intestinal barrier function and gut microbiota diversity in humans. Further, this study will likely advance our understanding of the relationship between intestinal permeability and cardiometabolic risk in humans. There is limited scientific evidence on the sustained effects of BFP in humans. To date our understanding is based on observational studies or acute studies in healthy participants who received once-off treatments [27,29,30]. The outcomes of the study may stimulate further research exploring the effects and mechanisms of action of BFP, including studies in populations with metabolic and other diseases.

A challenge will be the potential for participants to fall ill and require prescription medication such as antibiotics and anti-inflammatory medication. We used a 25% dropout rate to calculate the required sample size to accommodate for incidence of such illnesses. A further limitation presents the reliance of self-reported data such as health status, dietary intake and adherence to the dietary and lifestyle recommendations prior to the Lac/Man test, which may affect the study outcomes.

As the study population comprises relatively young individuals (aged between 25 and 55 years) without known diseases, this may not be representative of the general population. The selection criteria were chosen to minimise confounding factors due to the small sample size. A further limitation is that participants may be unaware that they have chronic diseases, for example HIV, which can affect intestinal permeability [61]. This study will rely on self-reported HIV status to exclude participants living with HIV, which may be prone to errors. Future studies can explore the effectiveness of BFP in a broader population including participants living with HIV, as well as in participants with metabolic diseases such as type 2 diabetes and hypertension.

## Supporting information

S1 File Protocol Baobab study v4. (PDF)

S2 File SPIRIT checklist Baobab Study. (PDF)

S3 File Human participant research checklist. (PDF)
