# Supplementary material for: Effects of Baobab fruit powder on gut and cardiometabolic health in obesity—Protocol for a randomised placebo-controlled trial
Source: PLoS One. 2025 Aug 13;20(8):e0328774. doi: 10.1371/journal.pone.0328774 (PMC12349125; doi:10.1371/journal.pone.0328774)
Supplement: S1 File — (PDF) [file pone.0328774.s001.pdf]

Protocol Title:

Effects of Baobab fruit powder on gut and cardiometabolic health in obesity – a randomised placebo-controlled trial

Short title: Baobab Study

Principal Investigators:

Prof Joel Dave (Division of Endocrinology, Department of Medicine, University of Cape Town, Groote Schuur Hospital, Cape Town, South Africa)

Dr Sylvia Riedel (Biomedical Research and Innovation Platform, South African Medical Research Council, Tygerberg, South Africa; Centre for Cardio-metabolic Research in Africa, Division of Medical Physiology, Faculty of Medicine and Health Sciences, Stellenbosch University, Cape Town, South Africa)

Prof Julia Goedecke (Biomedical Research and Innovation Platform, South African Medical Research Council, Tygerberg, South Africa; Health through Physical Activity, Lifestyle and Sport Research Centre (HPALS), FIMS International Collaborating Centre of Sports Medicine, Division of Physiological Sciences, Department of Human Biology, Faculty of Health Sciences, University of Cape Town, Cape Town, South Africa)

Co-Investigators:

Caroline D'Alton (Division of Physiological Sciences/Division of Sports Medicine, Department of Human Biology/Department of Family, Community and Emergency Care, University of Cape Town

Amy Mendham (Riverland Academy of Clinical Excellence, Riverland Mallee Coorong Local Health Network, South Australia Health, Australia; Health through Physical Activity, Lifestyle and Sport Research Centre (HPALS), FIMS International Collaborating Centre of Sports Medicine, Division of Physiological Sciences, Department of Human Biology, Faculty of Health Sciences, University of Cape Town, Cape Town, South Africa)

Pieter Venter (Biomedical Research and Innovation Platform, South African Medical Research Council, Tygerberg, South Africa)

Carmen Pheiffer (Biomedical Research and Innovation Platform, South African Medical Research Council, Tygerberg, South Africa; Department of Obstetrics and Gynaecology, Faculty of Health Sciences, University of Pretoria, Pretoria, South Africa)

Rabia Johnson (Biomedical Research and Innovation Platform, South African Medical Research Council, Tygerberg, South Africa; Centre for Cardio-metabolic Research in Africa, Division of Medical Physiology, Faculty of Medicine and Health Sciences, Stellenbosch University, Cape Town, South Africa)

Nonhlanhla Yende-Zuma (Biostatistics Research Unit, South African Medical Research Council, Tygerberg, South Africa)

Christo Muller (Biomedical Research and Innovation Platform, South African Medical Research Council, Tygerberg, South Africa; Centre for Cardio-metabolic Research in Africa, Division of Medical Physiology, Faculty of Medicine and Health Sciences, Stellenbosch University, Cape Town, South Africa; Department of Biochemistry and Microbiology, University of Zululand, KwaDlangezwa, South Africa)

Funding:

- SR received funding through the Access and Benefit Sharing (ABS) Compliant Bio-trade in South(ern) Africa (ABioSA) project Phase II, which is implemented by Deutsche Gesellschaft für Internationale Zusammenarbeit (GIZ) GmbH (Grant agreement 81291535).
- SR and JHG received baseline funding and in-kind contributions through SAMRC
- JHG received baseline funding through UCT

1. Table of Contents

2. Background and Significance 4
3. Study Aims and Objectives....6
   - 3.1 Aim....6
   - 3.2 Objectives 6
   - 3.3 Hypotheses: 6
4. Methodology....7
   - 4.1 Study design....7
     - 4.1.1 Participant recruitment, screening and randomisation 10
     - 4.1.2 Intervention 11
     - 4.1.3 Sample collection pre- and post- intervention 13
     - 4.1.4 Biochemical analyses 16
     - **Urinary Lac/Man ratio** 16
     - **Faecal SCFA**....16
     - **Blood analyses**....17
     - **Faecal microbiota analysis**....17
   - 4.2 Storage of samples for future use 18
   - 4.3 Statistical Analysis....18
     - 4.3.1 Sample size determination 18
     - 4.3.2 Statistical analysis 18
   - 4.4 Anticipated challenges....19
5. Management of the study 19
   - 5.1 Day to day management....19
   - 5.2 Team, expertise and responsibilities 20
     - 5.2.1 Data safety and progress monitoring 21
     - 5.2.2 Monitoring 21
   - 5.3 Facilities and expertise 22
6. Ethical considerations 22
   - 6.1 Ethical review....22
   - 6.2 Informed consent 23
   - 6.3 Confidentiality and anonymity 23
   - 6.4 Benefits and risks to participants 24
     - 6.4.1 Potential Benefits....24
     - 6.4.2 Potential Risks and discomfort 25
   - 6.5 Use of the results/impact 26

7. References 27
8. Annexures 33
8.1 Annexure – Information Sheet and informed consent forms 33
8.2 Annexure - Steering Committee Charter v1 October 2023 45

2. Background and Significance

Baobab (*Adansonia digitata* L.) is an iconic tree indigenous to the woodlands, savanna and grasslands of sub-Saharan Africa. Baobab leaves, seeds, bark, roots and fruit pulp are traditionally used and/or consumed throughout sub-Saharan Africa due to their nutritional value and anecdotal health benefits (Chadare *et al.*, 2009; Rahul *et al.*, 2015). In 2008, Baobab fruit powder (BFP) was authorised as a novel food ingredient and has since been commercially available in the European Union (EU) (document number C(2008)3046; http://data.europa.eu/eli/dec/2008/575/oj). The United States Food and Drug Administration (FDA) has classified BFP as "generally regarded as safe" (GRAS, GRAS notice 273, https://www.cfsanappsexternal.fda.gov/scripts/fdcc/index.cfm?set=GRASNotices&id=273). The BFP is also commercially available in health shops and supermarkets in South Africa. The powder typically contains significant levels of soluble fibre, polyphenols, vitamin C, citric acid and minerals such as calcium, magnesium, potassium and iron (Nicol, 1957; Eromosele *et al.*, 1991; Lamien-Meda *et al.*, 2008; Chadare *et al.*, 2009; PhytoTrade Africa, 2009; Ismail *et al.*, 2019; Khoja *et al.*, 2021; Foltz *et al.*, 2021).

Despite its commercial availability, its high fibre and polyphenol content and anecdotal health benefits, only a few studies have explored potential effects of BFP in humans. To our knowledge there is only one study that reported potential pre-biotic effects of BFP, showing increased production of short chain fatty acids (SCFA), such as acetate, propionate and butyrate, in faecal cultures from three human donors *in vitro* (Foltz *et al.*, 2021). Based on the high fibre and polyphenolic content of BFP, these pre-biotic properties may likely also affect intestinal barrier function, microbiota composition and metabolite production *in vivo*.

Increased intestinal permeability is now a well-established factor implicated in the pathophysiology of metabolic diseases such as obesity and type 2 diabetes, as well as cardiovascular disease (Cani *et al.*, 2007; Lassenius *et al.*, 2011; Camilleri, 2019; Khoshbin & Camilleri, 2020; Riedel *et al.*, 2022). The underlying mechanism linking increased intestinal permeability with metabolic diseases is likely the translocation of sub-clinical levels of microbiota-derived products such as lipopolysaccharide (LPS) from the intestinal lumen into the blood stream. Circulating LPS can activate immune responses resulting in low-grade inflammation characterised by increased levels of C-reactive protein and cytokines such as tumour necrosis factor alpha (TNFα) and interleukin-6 (IL-6) (Cani *et al.*, 2007; Camilleri, 2019). Importantly, interventions such as drugs or herbal formulations with intestinal barrier-protective properties have been shown to improve intestinal permeability (Kato *et al.*, 2017; Ried *et al.*, 2020).

Intricately linked with intestinal permeability and homeostasis are commensal microbiota composition and diversity, which are also decreased and/or altered in obesity and diabetes (Brunkwall & Orho-Melander, 2017; Dugas *et al.*, 2018; Cani, 2019; Delzenne *et al.*, 2019). While microbiota changes in obesity are widely reported in animal and human studies, there is not yet consensus regarding a clearly defined and generally recognised pattern of changes in microbiota that is associated with obesity or type 2 diabetes, presumably due to large inter-individual differences, the significant influence of host genetic and environmental factors, such as diet,

and the fact that what constitutes "healthy microbiota" is insufficiently defined (Fan & Pedersen, 2021; Olofsson & Bäckhed, 2022). However, literature suggests the diversity and richness decreases in obesity (Sze & Schloss, 2016) and changes in individual bacterial species modulates the availability of active metabolites such as SCFA and amino acids (Liu *et al.*, 2017), which influence intestinal barrier function and may be responsible for the observed metabolic effects (Fortea *et al.*, 2021). Diet, and specifically dietary fibre, profoundly affect microbiota composition through selection of saccharolytic bacterial strains that utilise and digest fibres (Makki *et al.*, 2018; Xu *et al.*, 2022) and several studies have shown that dietary fibre supplementation can affect microbial composition as well as intestinal barrier function in humans (Machado *et al.*, 2021; Deehan *et al.*, 2022; Zhang *et al.*, 2022; Roach *et al.*, 2022). Scientific data predominantly emanates from developed countries or the "Global North", leaving gaps in our knowledge with regards to microbiota composition, diversity and function in African and South African populations, which display significant differences due to factors such as genetic as well as cultural and dietary diversity (Allali *et al.*, 2021; Makhalanyane *et al.*, 2023; Ecklu-Mensah *et al.*, 2023).

Improved intestinal permeability and microbial composition may modulate dysglycaemia and possibly weight gain. The few studies involving human participants showed acute effects of Baobab fruit extracts on lowering postprandial glycaemia in healthy adults (Coe *et al.*, 2013; Rita *et al.*, 2022), while in a follow-up study, Coe & Ryan (2016) reported improved insulin sensitivity after consumption of white bread containing a Baobab fruit extract. Garvey *et al.* (2017) investigated the effect of BFP on satiety and reported decreased hunger after consumption of a smoothie containing BFP compared to a control. It is tempting to speculate that BFP, through its effects on intestinal permeability and microbial composition, may indirectly and/or directly impact on cardiometabolic risk by decreasing glucose absorption or modulating gut hormone release (Drucker, 2007; Mirzababaei *et al.*, 2022).

Based on the reports on the pre-biotic properties in conjunction with the high fibre and polyphenolic content of BFP, we hypothesise that BFP will decrease intestinal permeability, increase intestinal microbial diversity and subsequently lead to improvements in cardiometabolic risk factors.

This study will be the first to determine potential effects of BFP on intestinal permeability, and secondarily on gut microbiota and cardiometabolic risk factors in humans. In addition, the safety of BFP in humans will also be assessed. If effects on intestinal permeability, gut microbiota and/or cardiometabolic risk factors are detected, this may stimulate further research exploring the mechanisms of action of BFP in larger studies and in disease settings such as type 2 diabetes.

3. Study Aims and Objectives

3.1 Aim

Perform a randomised double-blind placebo-controlled trial to examine the effects of consumption of BFP on intestinal permeability in participants with obesity. As secondary aim we will also assess the effects of BFP on gut microbiota and cardiometabolic risk factors, as well as confirm the safety of BFP in humans.

3.2 Objectives

The objectives of this study are to measure the following changes in response to the 45-day BFP intervention compared to the control group:

- changes in intestinal permeability using the urinary lactulose/mannitol (Lac/Man) ratio (primary outcome)
- changes in blood biomarkers of intestinal permeability (secondary outcomes)
- changes in the composition and diversity of intestinal microbiota profiles using 16S ribosomal RNA gene sequencing (secondary outcomes)
- changes in cardiometabolic risk markers including anthropometric measurements (such as body mass index (BMI), waist and hip circumference), blood lipid profiles, fasting glycaemia, insulin resistance, inflammation and blood pressure (secondary outcomes)
- changes in liver (aspartate transaminase (AST), alanine transaminase (ALT), alkaline phosphatase (ALP), gamma glutamyl transpeptidase (GGT)) and kidney (creatinine) function and gastro-intestinal symptoms (safety outcomes)

3.3 Hypotheses:

The 45-day intervention with BFB will result in

- A reduction in the intestinal permeability as measured by the Lac/Man ratio and/or by a reduction in blood biomarkers
- An increase in alpha and beta diversity of microbiota (Shannon, Chao 1 index and/or Bray Curtis index, respectively) and increased abundance of SCFA-producing species.

We further hypothesize that improvement in markers of intestinal permeability following consumption of Baobab fruit powder for 45 days will result in

- A reduction in cardiometabolic risk markers characterised by reduced BMI, glucose, insulin, lipids, inflammatory markers and/or blood pressure.

Finally, based on the fact that BFP has been consumed in Africa for centuries, that it is safe for consumption in humans.

4. Methodology

4.1 Study design

The overview of the study design is described in Figure 1. In this randomised double-blind, placebo-controlled trial, 50 participants (women and men) with obesity will be randomised into an experimental (BFP) and a control (placebo) group. The BFP group will consume 16 g BFP daily for 45 days, while the control group will consume an isocaloric placebo containing 13.5 g tapioca flour with a similar appearance and taste to the BFP.

Prior to and following the 45-day intervention, participants will complete one testing session, as outlined in Table 1, during which the primary and secondary outcomes as well as potential confounders will be measured. In brief, intestinal permeability will be measured using the urinary Lac/Man ratio at 5 and 24 hours representing small intestinal and colon permeability, respectively. A stool sample will be collected for the measurement of composition and diversity of gut microbiota and to measure microbial metabolites that influence intestinal permeability and metabolism, such as SCFA. Blood biomarkers of intestinal permeability will be quantified. In addition, cardiometabolic risk factors will be assessed including anthropometric measurements (BMI, waist and hip circumference), fasting glycaemia and insulin resistance (Homeostatic Model Assessment for Insulin Resistance (HOMA-IR)), inflammatory markers (e.g. high sensitivity C-reactive protein (hs-CRP)), lipid profile, and blood pressure. Questionnaires will be administered to gain information on dietary intake, physical activity and health and lifestyle behaviours. To assess safety of BFP, liver (liver enzymes) and kidney (estimated glomerular filtration rate, GFR) function will be assessed at baseline, 2-weeks into the intervention and following the 45-day intervention. Gastrointestinal symptoms will be assessed bi-weekly using validated questionnaires, and side effects and any adverse events will be assessed weekly and when they occur.

The participants will be encouraged to maintain their usual dietary and lifestyle behaviours during the intervention period. Participants will be monitored weekly by alternating telephonic contact and visits at the research facility to confirm compliance, acceptability and tolerability. Adverse events, such as allergic reactions, and incidence of other potentially confounding health issues such as illnesses that require extended use of medication will also be assessed. Participants will be reimbursed for their time and travel costs.

Ethical approval was conditionally granted by the Human Research Ethics Committees (HREC) at the SAMRC on the 8th of May 2023. The study will be performed in accordance with the principles of South African Good Clinical Practice (SA GCP 2020 version 3), the Declaration of Helsinki (1964, as amended in Fortaleza Brazil, 2013), the ICH Good Clinical Practice (GCP) and the applicable laws of South Africa. Participants will be required to provide written informed consent prior to participation in the screening and the research study.

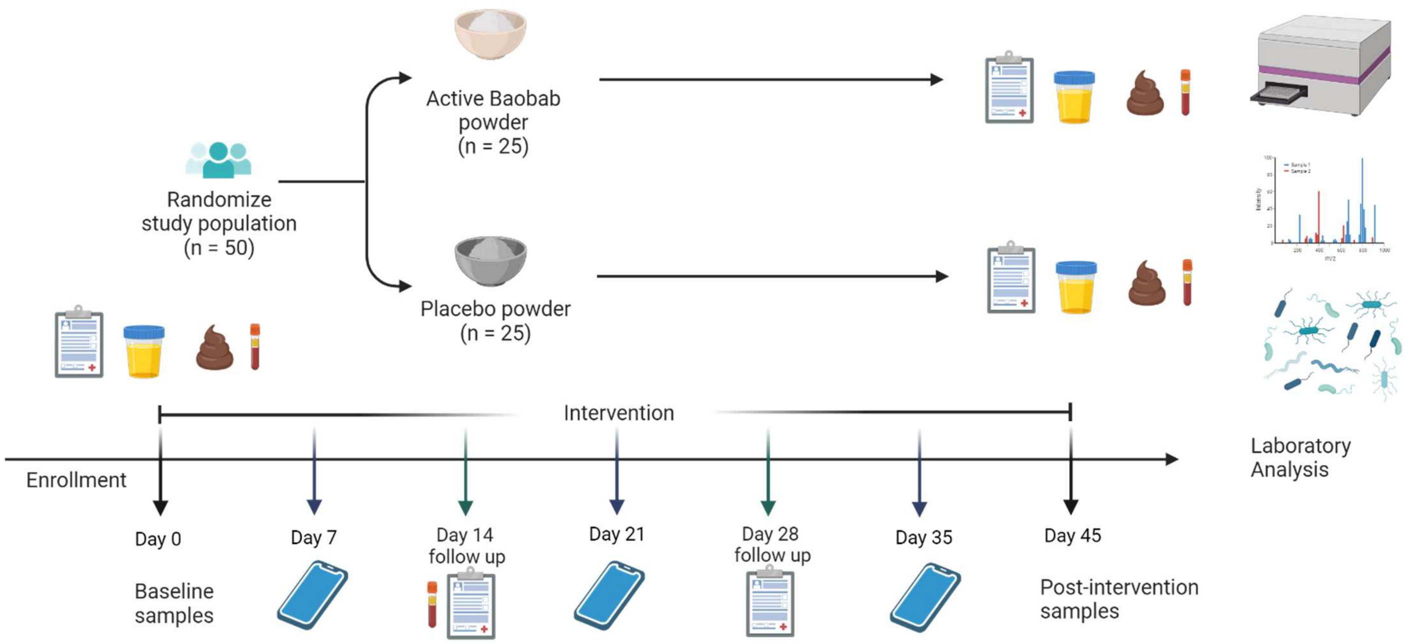

Figure 1 Study outline and sampling schedule for the proposed randomised placebo-controlled trial to determine intestinal permeability, gut microbiota and cardiometabolic risk factors following consumption of Baobab fruit powder (BFP) in participants (women and men) with obesity. Participants meeting the inclusion criteria will be invited to attend the baseline testing session where a urinary intestinal permeability test (Lactulose/mannitol test) will be conducted. Socio-demographic, health, physical activity, and dietary data will be entered directly into the electronic data capturing tool (REDCap). Anthropometric (BMI, waist and hip circumference) and blood pressure measurements will also be entered into REDCap. Fasting blood samples will be taken for determining safety outcomes (liver and kidney function), as well as blood biomarkers of intestinal permeability and inflammation, fasting glycaemia, insulin resistance and lipid profiles. A stool sample will be requested for analysis of microbiota composition and diversity. The BFP group will consume 16 g of BFP and the control group will consume a placebo (tapioca flour) with similar taste and caloric content daily for 45 days. The testing procedures will be repeated at the 45-day follow up visit. Participant compliance, tolerability, acceptability and symptoms will be monitored weekly through alternating telephonic contact and visits at the research facility. (Figure created with BioRender.com)

Table 1 Measurements at baseline and follow up at 45 days:

Outcomes to be measured	***Test***	***Sample type***
Primary outcome		
Intestinal permeability	• Lac/Man ratio (LC/MS)	Urine
Secondary outcomes		
Intestinal permeability	• LPS levels (ELISA)<br>• Soluble cluster of differentiation (sCD)14 (ELISA)<br>• Intestinal fatty acid binding protein (IFABP) (ELISA)<br>• LPS-binding protein (LBP) (ELISA)<br>• Kynurenine/tryptophan ratio (LC/MS)	Fasting blood
Microbiota	• Composition and diversity (16S rRNA metagenomic sequencing)<br>• SCFA (LC/MS)	Stool
Cardiometabolic risk factors	• Anthropometry (Weight, BMI, waist and hip circumference, waist/hip ratio)<br>• Fasting plasma glucose and insulin concentrations<br>• Serum lipid profile: triglycerides; high-density lipoprotein (HDL)-cholesterol and low-density lipoprotein (LDL)-cholesterol<br>• Inflammation markers (e.g. hs-CRP)<br>• Blood pressure	 Fasting blood<br>Fasting blood<br>Fasting blood
Safety outcomes	• Liver function test (AST, ALT, ALP, GGT)<br>• Kidney function test (creatinine)<br>• Gastro-intestinal symptoms questionnaire	Fasting blood<br>Fasting blood
Covariates/confounders		
	• Dietary intake (estimated 3-day food record at the pre-intervention testing session and at the post-intervention testing session)<br>• Physical activity (global physical activity questionnaire (GPAQ))<br>• Health and lifestyle questionnaire (previous smoking, drinking habits, allergies, food intolerances, stool habits (Bristol stool chart, gastrointestinal symptoms questionnaire), supplement use, socio-demographics, perceived stress)	
Future analysis for stored samples will include:		
	• Markers of intestinal permeability and inflammation	Stool
	• Circulating inflammatory markers such as cytokines<br>• Circulating markers of cardiometabolic risk<br>• Circulating microbial biomarkers and metabolites<br>• Transcriptomics to determine underlying mechanisms related to intestinal permeability and cardiometabolic risk	Fasting blood<br><br>Fasting blood collected in RNA Paxgene tubes

4.1.1 Participant recruitment, screening and randomisation

We will advertise through UCT and SAMRC mailing lists, through social media (Facebook, LinkedIn and Twitter) and on the SAMRC website. Awareness of the study will be raised through distribution of a flyer as advertisement at UCT and SSISA and the surrounding community to attract participants that are living close to the research facility. We also summarised the study procedures in a participant leaflet. Sample collection will be conducted at the Health through Physical Activity, Lifestyle and Sport Research Centre (HPALS), Division of Physiological Sciences, Department of Human Biology, University of Cape Town (UCT), which is based at the Sports Science Institute of SA (SSISA) in Newlands. Prior to recruitment into the study, participants will undergo screening to confirm their eligibility.

Inclusion criteria:

- Ability to sign informed consent
- Age between 30 and 45 years
- BMI ≥ 30 kg/m^2
- Weight stable (less than 5 kg weight change in the last 6 months)

Exclusion criteria:

- History of gastrointestinal diseases (e.g. irritable bowel syndrome, coeliac disease, Crohn's disease, Ulcerative Colitis) and gastrointestinal surgery
- Current smoker (including e-cigarettes) or user of chewing tobacco
- Current bacterial or viral infection
- Pregnancy or breastfeeding
- Regular use of chronic (defined as either daily or regular intake at least 3 times per week) medication (prescription medication for obesity, chronic non-communicable and infectious diseases, immunosuppressants, frequent use of over-the-counter drugs such as non-steroidal anti-inflammatory drugs and laxatives)
- Known chronic diseases (with or without treatment with prescription medication) or screen-detected diseases, e.g.:
    - autoimmune diseases
    - hypertension (BP >140/90 mm Hg);
    - Type 1 or Type 2 diabetes (FBG > 7 mmol/l)
    - dyslipidaemia (TG >1.7 mmol/l, LDL >3.0 mmol/l)
    - heart disease
    - stroke
    - reproductive diseases (e.g. endometriosis or polycystic ovary syndrome)
    - thyroid diseases
    - infectious diseases

- Severe allergies, known allergy to baobab (Martini *et al.*, 2019) or tapioca flour, and food intolerances (e.g. sugar alcohol intolerance)
- Oral antibiotic use, fibre, pre- and/or probiotic supplement use in the preceding 3 months (Seethaler *et al.*, 2021)

The contents of the intervention and placebo sachets will have non-identifiable labels and will be packaged in foil sachets protected from light so that study staff and participants will be blinded to the group allocation. Participants will be randomised at a 1:1 ratio using block randomisation with random permuted block sizes of 2 and 4, which will be completed by the study statistician. This will reduce bias and achieve balance in the allocation of participants to treatment arms. A pharmacist at the Clinical Research Centre at UCT will be responsible for collection, storage and dispensing of the sachets.

4.1.2 Intervention

Baobab fruit powder is a product commercially available in South Africa and will be supplied by the African Baobab Alliance (ABA), which is a not-for profit sector organisation representing multiple stakeholders in the Baobab sector, including Baobab fruit harvesters from underserved rural Southern African communities of which the majority (80 %) are women. The ABA approached SAMRC scientists to design a research project on investigating effects of BFP on the gut, but did not participate in design of the trial, which was guided by the scientific input by the researchers.

The BFP provided for this study is in its natural form as it is available on the shelves in South African supermarkets. It is produced from fruit that are free harvested by rural harvesters in Limpopo and the powder used for this trial will be sourced from EcoProducts through the African Baobab Alliance. EcoProducts is located in Makhado, Limpopo province, South Africa. Baobab fruit have a hard shell which protects the seeds that are covered in fruit powder. This powder is called the fruit pulp or fruit powder. The processing includes opening the fruit and separating the seeds from the powder. The fruit powder is further milled, sieved and then packaged by EcoProducts, Ltd in Makhado, South Africa. Tapioca flour will be purchased from Nature's Choice (Cape Town, South Africa) and both BFP and placebo will be packaged into single serving sachets by a local packaging company (JoyPak, Cape Town, South Africa). The sachets will then be transported to the registered Research Pharmacy at the Clinical Trials Centre at UCT, where a pharmacist will label active and placebo sachets according to regulatory requirements for use in human clinical trials.

The BFP (16 g, 983 kJ/100 g) and placebo will be packaged in identical single serving foil sachets protected from light to be consumed daily for a period of 45 days. A placebo will be prepared with a similar appearance and taste to the BFP containing tapioca flour (12 g, 1348 kJ/100 g) and citric acid (1.5 g) to yield equal caloric content (157 kJ or 38 kcal, which is equivalent to half a slice of bread). However, previous studies using Baobab did not use placebos (Coe *et al.*, 2013; Coe & Ryan, 2016; Garvey *et al.*, 2017; Rita *et al.*, 2022), were observational is design (Ahmed *et al.*, 2022) or delivered Baobab as a drink (Evang *et al.*, 2021). There is currently no published

study on BFP with a similar design on which to base the placebo formulation. The sachet contents can be suspended in up to 1 L of water or added to other cold beverages such as smoothies, juice or to cereals etc. The BFP should be consumed daily and should not be heated.

The 16 g dose was selected based on typical Baobab products on the market, which suggest servings of between 8 and 24 g per day. Our study intervention is equivalent to 2 heaped tablespoons and will deliver approximately ~8 g of dietary fibre per day. In addition, previous studies reported long-term use (tolerability) (Evang *et al.*, 2021), palatability (Garvey *et al.*, 2017), significant changes in postprandial glycaemia (Coe *et al.*, 2013; Rita *et al.*, 2022) and insulin response (Coe & Ryan, 2016) with dosages of 16 g and above. The duration (45 days) was chosen based on previous studies (Del Piano *et al.*, 2014; Kato *et al.*, 2017) reporting statistically significant differences in the Lac/Man ratio between the respective intervention and placebo-treated groups.

The risks involved in consuming Baobab fruit powder and the Placebo (tapioca flour) are minimal, when taking into consideration that communities in Africa have incorporated Baobab fruit powder into their diets for centuries (Asogwa *et al.*, 2021; Komane *et al.*, 2023) and further, tapioca flour is used as a thickening agent in many foods, with minimal risk. There is some data available from previous studies where participants consumed Baobab fruit powder for extended periods, even though these studies tested different hypotheses. For instance, Ahmed *et al.* (2022) recruited participants that consumed Baobab fruit juice over a period of several years (self-reported) and they compared lipid profiles as well as kidney and liver markers between Baobab consumers and controls. While no differences in blood glucose, liver and kidney markers were evident, the authors also did not report any adverse events. Furthermore, Evang *et al.* (2021) supplied school children with a drink containing 20 g BFP daily for 83 days with the aim to improve iron deficiency. These authors reported no adverse events; however, they did also not show improvement in iron status. A similar study conducted in Nigeria also found no adverse event but reported improved iron status (Nnam *et al.*, 2011).

To ensure close follow up regarding compliance, palatability and tolerability (Lambert *et al.*, 2007) of the intervention, the participants will be asked to attend the research facility at HPALS at UCT every two weeks (on day 14 and 28). At these visits, additional data such as body weight, blood pressure and health information relating to symptoms and/or illnesses will be collected. This will include numerical scores for the Bristol stool chart (values between 1 and 7) and gastrointestinal (GI) symptoms such as nausea, GI rumblings, abdominal pain, bloating, flatulence, and diarrhoea rated on a 4-point scale (none, no more than usual, somewhat more than usual, much more than usual) (Stewart *et al.*, 2018; Deehan *et al.*, 2020) and the score will used for assessing safety outcomes throughout the trial period.

Participants will also be contacted telephonically every other week to monitor compliance, symptoms and tolerability. During the weekly contact, information about adverse events, such as allergic reactions, and incidence of other potentially confounding health issues such as illnesses that would require extended use of medication, will be collected. Participants are instructed to immediately inform the trial team should they encounter an adverse reaction, such as an allergic reaction to the intervention, and will be referred to the trial

physician for medical assessment and treatment. Should participants develop an illness that requires prescription of medication such as antibiotics, the case will be assessed by the trial steering committee and an assessment will be made about the exclusion of the participant. Should a participant stop taking the intervention, the participant will be requested to describe the reasons, and potential exclusion will also be assessed by the trial steering committee.

Compliance will be checked by counting the returned empty packaging. Participants will be provided with sachets containing BFP and placebo sufficient for 14 days at the initial testing session and on days 14 and 28 when they visit HPALS at UCT. Study staff will obtain contact information for appointment reminders and follow-up appointments. Participants will be compensated (R300, R50 x 6 hours) for their time for test sessions where Lac/Man test, blood draw, anthropometry and interviews are conducted and R35 (1 hour) for the initial screening and subsequent monitoring visits at the research facility on day 14 and 28. Travel expenses will be reimbursed at R100 per visit. Participants will receive refreshments after the Lac/Man and blood test visits. Participant compensation aligns with the recommendations stipulated by the South African Health Products Regulatory Authority (https://www.sahpra.org.za/wp-content/uploads/2022/01/SAHPGL-CEM-CT-02_v2-Guideline-for-CT-Participant-Time-Inconvenience-and-Expense-TIE-Compensation-Model.pdf).

4.1.3 Sample collection pre- and post- intervention

Screening visit:

Volunteers that meet the inclusion/exclusion criteria will be invited to attend a screening visit where the purpose of the study and the procedures will be explained in detail. Participants will receive the information leaflet and be given the opportunity to ask questions and asked to sign the informed consent form if they agree to participate in the study. Participants will be able to take the informed consent form home to consider and decide about participation in the study in their own time.

Anthropometric measurements including height and weight, in lightweight clothing without shoes will be determined using standard procedures. An electronic weight scale will be used to measure weight to the nearest 0.1 kg (Tanita TBF-410GS Total Body Composition Analyzer, Tokyo, Japan) and height will be measured to the nearest 0.1 cm using a wall-mounted stadiometer (Holtain, Ltd., Crosswell, UK) (Goedecke *et al.*, 2018; Ratshikombo *et al.*, 2021).

Systolic and diastolic blood pressure will be measured on the left arm using a digital blood pressure monitor (Omron M6, Kyoto, Japan) and appropriate cuffs. After the participant had been seated for at least five minutes, three blood pressure readings will be taken at two-minute intervals. For each participant, the average of the second and third readings will be used in the analyses.

At this visit, once study participants have completed the screening questionnaire satisfactorily, i.e. complying with all other exclusion criteria, a fasting venous blood (~8 ml) will be drawn into SST and FO tubes by a trained phlebotomist for the subsequent determination of plasma glucose and serum lipid profile (HDL-cholesterol,

LDL-cholesterol, total cholesterol and triglycerides). These samples will be sent to the National Health Laboratory Services (NHLS, Groote Schuur, Cape Town) for immediate analysis. The results of these tests as well as the BMI and blood pressure measures, will be sent to the study physician for review and comment and then given to the participants by the study staff. Those participants that are identified as having dyslipidaemia (Triglycerides > 1.7 mmol/L, LDL > 3.0 mmol/L) or diabetes (fasting plasma glucose > 7.0 mmol/l) or hypertension (BP > 140/90 mm Hg) at screening or at any time during the study will be immediately referred by the study physician to their Community Healthcare Centres (CHCs) for early initiation of treatment by doctors at the CHCs. The doctors at the CHC's will manage the participants according to the Practical Approach to Care Kit (Primary Care Guide for the Adult, Western Cape Edition). Participants with dyslipidaemia, hypertension and/or diabetes will be excluded from the study.

Test sessions at the research facility:

Participants that are eligible for the study and have signed the informed consent form will be given the EasySampler kit to take home for the collection of a pre-intervention stool sample for microbiota analysis. The use of the kit will be explained in detail verbally and written instructions are included in the kit. The participants will be asked to store the stool sample in their fridge at home (at 4° C in a sample tube) and delivered to the research facility within 6 hours. The participant will be asked to bring along the stool sample to the research facility. Transport of the sample at room temperature will not affect its integrity. At the research facility, samples will be aliquoted and stored frozen at -80° C in the laboratory for long-term storage and prior to microbial DNA extraction and SCFA analysis. Participants will be provided the EasySampler kit for the post-intervention stool sample at the follow up visit on day 28. The post-intervention stool sample will be requested before or on the post-intervention screening session.

Participants will be invited to attend one 6-hour testing session prior to and one 6-hour testing session on completion of the 45-day intervention at HPALS at UCT. Participants will be asked to avoid strenuous exercise, alcohol, non-nutritive sweeteners (e.g. in chewing gum), spicy food, caffeine (including tea), sporadic over-the-counter medication (such as headache pills or other nonsteroidal anti-inflammatory drugs) and supplement use for at least 48 hours prior to and for 24 hours following the testing session. On these testing days, participants will be asked to attend the research facility following a 10-12 hour overnight fast.

Procedures during the testing session:

- The intestinal permeability Lac/Man test will be conducted as described by Seethaler *et al.* (2021). A urine sample will be collected prior to the test to account for baseline levels of mannitol and lactulose. Lactulose (5 g, Dulphalac, Clicks) and 2 g mannitol (Merck Life Science) will be dissolved in 200 ml water to be consumed completely within 5 minutes by the participants to initiate the test procedure. Cumulative urine will be collected for 24 hours, of which the first 5 hours will be collected at the research facility (small intestinal permeability). Participants will receive water after 2 hours and will be provided with a meal after 5 hours whereafter they can leave the research facility. Participants will be provided

with one litre containers and a funnel to collect urine over the 24 h period. The 24 h urine samples will be collected from the participants home on the following day. Total urine volumes will be recorded, and aliquots will be stored frozen at -80 °C before liquid chromatography/mass spectrometric (LC/MS) for the quantification of lactulose and mannitol.

- At the pre-intervention session, fasting venous blood (~45 ml) will be drawn into SST, FO, RNA PAXgene® and EDTA tubes by a trained phlebotomist for the subsequent determination of serum insulin, inflammatory markers (e.g. hs-CRP) and biomarkers of intestinal permeability (e.g. LPS, LBP, sCD14, IFABP) as well as safety endpoints (creatinine, AST, ALT, ALP, GGT). The SST, FO and EDTA tubes will be centrifuged, and serum and plasma aliquots stored at -80°C until further analyses. The sample collected in RNA PAXgene® tubes will be stored for future transcriptomic analyses for which ethical approval will be sought separately.

- At the 2 week follow up session, fasting venous blood (~10 ml) will be drawn into SST tubes by a trained phlebotomist. The tubes will be collected by NHLS for determination of the safety endpoints (creatinine, AST, ALT, ALP, GGT).

- At the post-intervention testing session, fasting venous blood (~45 ml) will be drawn into SST, FO, RNA PAXgene® and EDTA tubes by a trained phlebotomist for the subsequent determination of plasma glucose, and serum insulin, lipid profile (HDL-cholesterol, LDL-cholesterol, total cholesterol and triglycerides), inflammatory markers (e.g. hs-CRP) and biomarkers of intestinal permeability (e.g. LPS, LBP, sCD14, IFABP) as well as safety endpoints (creatinine, AST, ALT, ALP, GGT). The SST, FO and EDTA tubes will be centrifuged, and serum and plasma aliquots stored at -80°C until further analyses. The sample collected in RNA PAXgene® tubes will be stored for future transcriptomic analyses for which ethical approval will be sought separately.

- Lifestyle, health, socio-demographic information such as age, gender, self-reported ethnicity, family history of disease, personal health, gastrointestinal symptoms and stool consistency (Bristol stool chart), health habits and stress, lifestyle factors (previous smoking, alcohol intake, physical activity) and socio-economic data (e.g. by assessing factors such as education level, occupation and employment, housing) will be collected using validated questionnaires (Goedecke *et al.*, 2018; Ratshikombo *et al.*, 2021) and entered directly into the electronic data capturing tool Research Electronic Data Capture (REDCap) (Harris *et al.*, 2009, 2019) using tablets. In addition, physical activity will be estimated using the validated Global Physical Activity Questionnaire (GPAQ) (Dickie *et al.*, 2014). Dietary intake will be estimated using an estimated 3-day food record, including 2 non-consecutive weekdays and one weekend day. The dietary assessment is focused on estimating total energy, macronutrient and fibre intake, and used as a covariate to confirm that the participants maintain their usual dietary intake (including fibre intake) over the 45-day intervention period. The participants will be trained on completing the food record during

the initial 6-hour testing session, including quantifying the types of food, preparation methods, and portion size estimation. The participants will be provided with a take-home guide to assist with the dietary record. The records will be checked and verified by the dietician/research assistant when the participant returns the dietary records at the bi-weekly follow-up visit. Nutrient intake will be converted to grams and analysed using the South African Food Composition Database System (SAFOOD, the South African Food Composition Database, South African Medical Research Council, Cape Town, South Africa) (Ratshikombo *et al.*, 2021).

- At the post-intervention testing session, anthropometric measurements including weight, in lightweight clothing without shoes, will be determined using standard procedures. An electronic weight scale will be used to measure weight to the nearest 0.1 kg (Tanita TBF-410GS Total Body Composition Analyzer, Tokyo, Japan) (Goedecke et al., 2018; Ratshikombo et al., 2021). Systolic and diastolic blood pressure will be measured on the left arm using a digital blood pressure monitor (Omron M6, Kyoto, Japan) and appropriate cuffs. After the participant had been seated for at least five minutes, three blood pressure readings will be taken at two-minute intervals. For each participant, the average of the second and third readings will be used in the analyses.

- At the pre-and post-intervention session, waist circumference will be measured in the mid–axillary line at the midpoint between the lower margin of the last palpable rib and the top of the iliac crest at the end of normal exhalation, and hip circumference at the largest protrusion of the buttocks, will be measured to the nearest 0.1 cm. Whole body composition will be estimated using a Quantum Legacy Bioelectrical Impedance Analyser (BIA, RJL Systems, USA).

4.1.4 Biochemical analyses

Urinary Lac/Man ratio

Ultra-high performance liquid chromatography (UHPLC, AB Sciex – EXIONLC 2.0+) coupled to a triple quadrupole mass spectrometer (MS/MS; AB Sciex Qtrap 6500+) will be used to determine the urinary Lac/Man ratio to evaluate the intestinal barrier function as described by Gervasoni *et al.* (2018). The UHPLC separation will be performed using an ACQUITY UPLC BEH amide column and sample preparation will be limited to a “dilute and shoot” method using labelled internal standards which will provide the highest reliability in terms of reproducibility and accuracy. Due to the low ionization efficiency of these sugars, a comparison between electrospray ionization (ESI) and chemical ionization (APCI) will be conducted.

Faecal SCFA

Short chain fatty acids in stool samples will be determined as described by Song *et al*. (2019). Briefly, stored faecal samples (-80°C freezer) will be thawed, dissolved in acetonitrile and derivatised prior to separation using an Agilent poroshell 120 EC-C18 Column and MS analysis performed on the AB Sciex Qtrap 6500+ operated in

the negative ionisation mode. The multiple reaction monitoring (MRM) transitions of all targeted compounds include the precursor ions and the signature product ions. The MS parameters such as declustering potential, collision energy and collision cell exit potential will be optimised to achieve the optimal sensitivity. SCFAs will be presented as individual SCFAs (µg/g), including butyric acid, propionic acid, acetic acid and valeric acid, as well as total SCFAs (sum of 4).

Blood analyses

One EDTA, FO and SST tube each will be sent to National Health Laboratory Services (Groote Schuur, Cape Town) for plasma glucose, insulin and serum lipids, inflammatory markers and safety markers (creatinine, ALP, AST, ALT, GGT). Briefly, plasma glucose will be determined using a colorimetric assay (Randox, Gauteng, South Africa) and serum insulin, lipid profile and high-sensitivity CRP will be measured using immunochemiluminometric assays (IMMULITE 1000 immunoassay system, Siemens Healthcare, Midrand, South Africa). LDL-cholesterol concentrations will be calculated using the Friedewald equation (Friedewald *et al.*, 1972). The HOMA-IR index will be calculated using fasting plasma glucose and insulin levels according to Matthews *et al.* (1985). The remaining SST and EDTA tubes will be centrifuged, and serum and plasma aliquots will be stored at -80 °C until further analyses and for long-term storage.

Biomarkers of intestinal permeability will be measured in plasma or serum samples in duplicate using commercially available Enzyme linked immunosorbent (ELISA) kits. The following targets were chosen for intestinal permeability and will be measured at baseline and following the 45-day intervention:

- plasma LPS levels will be assessed using a commercial kit (Life Technologies) and a plate reader (Spectramax i3x, Molecular Devices).
- serum LBP, sCD14 and IFABP will be measured using commercially available Sandwich ELISA assay kits (R&D Systems) using a plate reader (Spectramax i3x, Molecular Devices).
- the kynurenine/tryptophan ratio, which is an indicator of indoleamine 2,3-dioxygenase (IDO) activity, will be quantified. IDO is a key enzyme linking intestinal barrier function with gut microbial metabolites (Laurans *et al.*, 2018). Kynurenine and tryptophan will be measured using the LC/MS method described by Fuertig *et al.* (2016) in plasma samples.

Faecal microbiota analysis

Nucleic acids will be extracted using the MagMax Microbiome Ultra kit (Thermo-Fisher) on the Kingfisher robotic platform. PCR of the 16S rRNA V4-5 region will be performed and sequenced using 2 x 150 paired-end multiplexed sequencing on the NovaSeq 6000. The DNA extraction and 16S ribosomal RNA metagenomic sequencing will be conducted by Dr Jack Gilbert, Director of the Microbiome and Metagenomics Center, at the University of California, San Diego, USA. The raw data will be sent to SAMRC for analysis. All amplicon sequencing data will be analysed using the QIIME2 platform and made freely available to investigators through the Qiita database. For these data, all sequence data will be quality filtered and de-multiplexed, followed by

DeBlur amplicon sequence variant (ASV) calling to obtain unique sequences, which will be compared against various databases to allow for annotation.

4.2 Storage of samples for future use

Blood, urine and stool samples will be stored in aliquots -80 °C at HPALS at UCT until all samples have been collected and follow up visits are completed. The freezers are fitted with continuous temperature monitoring with an alert system. Thereafter they will be transported on dry ice to BRIP at the SAMRC for storage at -80 °C prior to biochemical analyses. Freezers at BRIP are also fitted with continuous temperature monitoring and an SMS alert system for laboratory managers.

Permission to store blood, stool and urine samples for future biomedical analyses, specifically exploring diseases such as gastro-intestinal diseases, diabetes, cardiovascular disease, will be obtained from the participants and documented on the informed consent form. New and additional research questions may arise from the planned analyses, which may become important for our understanding of the benefits of BFP. The participants will have the opportunity to select which samples may be stored and duration of storage in a separate informed consent. Approval from the Human Research Ethics Committee at the SAMRC and University of Cape Town will be obtained before any additional research is performed on stored blood, stool and urine samples.

4.3 Statistical Analysis

4.3.1 Sample size determination

The study is powered on the primary study endpoint, the Lac/Man ratio, which is based on the data reported by del Piano *et al.* (2014) for a similar treatment period. Considering a mean ± standard deviation (SD) difference in the Lac/Man ratio between treatment and control groups (0.015 ± 0.006 versus 0.021 ± 0.007) after the 45-day treatment (Del Piano *et al.*, 2014), significance level of $p < 0.05$ (two-sided test) and power of 80 %, 20 participants per group would be sufficient for our study to detect significant differences. When factoring in a drop-out rate of 25 % (5/25), 25 participants were selected per group.

4.3.2 Statistical analysis

Results will be presented as means ± SD or medians and interquartile ranges for normally and skewed data, respectively. Non-normally distributed data will be transformed for parametric analysis. Intention to treat analysis approach will be used. A Mixed Effects model will be used to compare within and between groups (control and BFP) differences in the change in primary and secondary outcome variables adjusting for potential confounders as outlined in table 1. Data will be analysed using NCSS 11 Statistical Software (NCSS, LLC. Kaysville, USA) and/or STATA SE (version 17, StatCorp, Texas, USA).

Statistical comparisons for taxonomic differences will be performed using Songbird (Morton *et al.*, 2019), an experimentally validated, compositionally coherent differential abundance method. Statistical visualization of

reference frames from Songbird will be performed using Qurro (Fedarko *et al.*, 2020), which forms part of a compendium of PCA/PcoA visualization tools such as EMPress (Cantrell *et al.*, 2021) and EMPeror (Vázquez-Baeza *et al.*, 2013), designed for the analysis of microbiome datasets. For longitudinal data we will employ volatility analyses, such as semivariograms (Gonzalez & Knight, 2012) and Compositional Tensor Factorization (Martino *et al.*, 2021), to decompose timeseries responses into components acting on different timescales. Using these approaches, we will identify taxa that are associated with Baobab fruit powder supplementation, faecal SCFA production, biomarkers of intestinal permeability (LPS, LBP, sCD14, IFABP) and cardiometabolic parameters including glucose, insulin, lipid profiles, inflammatory markers across intervention groups.

4.4 Anticipated challenges

It is possible that there will be a selection bias as generally women have higher tendencies to participate in research studies, however, every attempt will be made to recruit equal numbers of men and women, with adjustment for sex in the statistical analysis. A challenge will be the potential for participants to fall ill and require prescription medication such as antibiotics and anti-inflammatory medication. These participants will be excluded from the analyses. We used a 25 % dropout rate to calculate the required sample size to accommodate for incidence of such illnesses. In the unlikely event that there is a dropout of greater than 25%, we will recruit additional participants until we meet the required sample size.

A further limitation is that participants may be unaware that they have chronic diseases, for example HIV infection. For this study we will rely on self-reported HIV status to exclude participants living with HIV as it is known that HIV can affect intestinal permeability (Webel *et al.*, 2017). Future studies can explore the effectiveness of BFP in participants living with HIV, as well as in participants with metabolic diseases such as type 2 diabetes and hypertension.

5. Management of the study

5.1 Day to day management

Dr Riedel will be responsible for the overall management of the project, and oversee all procurement and financial aspects, sample collection, storage and analyses, and data management. Prof Goedecke will oversee the management and collection of the anthropometric, cardiometabolic markers, socio-demographic and lifestyle data. A registered research nurse will be responsible for drawing blood samples and the weekly assessment of compliance, symptoms and adverse events. Prof Dave will manage medical aspects and adverse events as the trial clinician and will be supported by Dr D'Alton at the research facility and when necessary. Samples will be stored at HPALS until recruitment and follow up visits are complete and will then be transported to BRIP, SAMRC on dry ice for analysis and long-term storage.

A trial committee will be responsible for overseeing trial safety and progress, adherence to the protocol and dropouts, consider new information and dissemination of the results.

Data will be collected and managed within RedCap. Tablets will be used for data collection and all data will be stored on a secure server by the EDMS service provider with respondents only being identified in the system with a barcode. At the end of the trial, the data will be downloaded from the EDMS and once it is cleaned and approved by the PI an instruction will be given to the EDMS service provider to delete it from their server.

Training of students

Two MSc students and one PhD student will be recruited to the project. Data collection will be performed by the students under the close supervision and training of the research team. Prior to initiating the study, students will receive comprehensive training on all procedures regarding anthropometry, cardiometabolic markers, the Lac/Man test and the questionnaires. Prof Goedecke and Dr Hoosen will provide training and oversee the dietary assessment (estimated food records and SAFOODS analysis). Training will also entail entering and anonymising participants personal and medical information. Staff and students will be trained to ensure that patient confidentiality is maintained at all stages of the data collection process. The project manager and a trained research assistant will be responsible for recruitment and retention of participants by managing calendar invites and reminders to the participants.

5.2 Team, expertise and responsibilities

Prof Dave will take full responsibility for the trial and delegate responsibilities to the research team. Specifically, he will oversee aspects regarding safety and potential adverse events.

Dr Riedel will assume full responsibility for managing the trial as well as the post-trial biochemical analyses and will ensure that the results will be disseminated as publications in high-impact peer-reviewed reputable international journals and presentations at local and international scientific conferences. Local media, including magazine, radio and newspaper articles, and social media, such as LinkedIn and Facebook with the guidance of the SAMRC's Corporate Communications Department, will be used to communicate results to the lay public.

Prof Goedecke will be the liaison with the research facility at HPALS at UCT and will oversee the management and collection of the anthropometric, cardiometabolic markers, socio-demographic and lifestyle data.

A registered nurse will be employed to assist with day-to-day management of the study, including assisting with recruitment and retention, collection of blood samples, administering the Lac/Man test, sample and data collection, manage sample storage, and monitor and manage adverse events.

Dr D'Alton will be the lead clinician at the trial site.

Dr Hoosen will supervise the quantitative and qualitative nutritional data collection, coding, cleaning and analysis and provide guidance and advice for the nutritional aspects of the project.

Dr Yende-Zuma will assume responsibility for statistical analyses. In addition, students and staff will also have the opportunity to be trained and supervised in the relevant statistical methods.

Dr Venter will be responsible for the development of the LC/MS methods, as well as analyses of samples, data analysis, student training and supervision, data interpretation, write up and reporting.

Prof Pheiffer will be responsible for guidance and data interpretation on markers of glucose homeostasis and metabolic risk, as well as student supervision, data interpretation and write up.

A data manager will be employed to set up and programme the REDCap database as well as for data management and weekly data quality control and assurance during participant recruitment.

Prof Dugas will be responsible for facilitating analysis and interpretation of microbiota composition and diversity, as well as for training of staff and students in the relevant analysis techniques.

Dr Mendham will be responsible for providing guidance and training of staff on the day-to day management of the participant recruitment, participant health screening, blood sample collection, data collection and research facility visits.

Prof Johnson will be responsible for guidance and data interpretation of cardiovascular markers in the study.

Prof Muller will consult with regards to interpretation of cardiometabolic risk markers and methodologies of microbiota analysis.

Our collaborator, the African Baobab Alliance will be responsible for supplying the BFP, however, the ABA was not involved in any aspects of the trial design.

5.2.1 Data safety and progress monitoring

The trial committee will internally monitor the recruitment progress, data collection, retention and other issues relating to participants and participant safety weekly.

The steering committee (SteerCo) will include Dr Riedel and Prof Goedecke, as well as four independent advisors with the relevant expertise. The committee will meet as outlined in the SteerCo charter v1 October 2023, to discuss progress, recruitment issues, data collection and adverse events (Annexure 8.2).

5.2.2 Monitoring

The HIV and other Infectious Diseases Research Unit (HIDRU), SAMRC will provide a monitor for the trial. A hybrid model may be followed including a combination of in-person and remote monitoring. Documents can be shared electronically with the monitor. Monitoring sessions will be scheduled once per month.

5.3 Facilities and expertise

The project will be run at the HPALS at UCT, at which the team has performed many intervention studies. Which is housed within the Sports Science Institute of South Africa. HPALS has teaching, clinical, exercise and biochemical research facilities, which are located on the 2nd and 3rd floor of the building. In addition, SSISA offers medical and allied health services, including sports doctors, dieticians, radiologists, psychologists etc on the ground and 1st floors, as well as houses a short-term stay hospital on the 5th floor. The HPALS UCT clinical research facility is based on the 2nd floor of the building and is strictly access controlled. There is a private waiting area, consultation rooms and clinical and biochemical laboratories that will be used for the study visits, with access to a kitchenette and toilet facilities for staff and participants. The site has all the required equipment to successfully complete the study including scales, stadiometers, digital blood pressure monitors, balances, temperature-controlled centrifuges, fridges, freezers, ice machines and glassware. Each floor of the building has an AED and emergency buttons linked to the doctors rooms, and there is also an emergency trolley available in the unlikely event of a medical emergency.

BRIP at SAMRC has fully functioning and equipped laboratories necessary for the successful sample analysis for this study. Equipment such as plate readers for the ELISA assays (SpectraMax i3x) and state-of-the art LC/MS system (AB Sciex 6500+) and electrochemiluminescence multiplex assays (MSD Meso Quickplex SQ 120 sector plate reader) are available in the laboratories of BRIP. BRIP is also equipped for high throughput liquid handling, including high-quality multi-channel and multi-dispensing tools to generate accurate, precise and reliable data. Research and data analysis expertise as well as skilled technical support is available at the SAMRC and within the project collaborators.

6. Ethical considerations

6.1 Ethical review

Provisional approval by the Human Research Ethics Committee of the South African Medical Research Council has been granted and we are currently awaiting final approval from HREC at UCT, which required us the determine whether SAHPRA approval is required for this study. Further approval as relevant by the equivalent bodies at the institutions of the co-applicants or students will be sought where it is a requirement for their involvement in the study. The study will be conducted following the principles outlined in SA GCP 2020 version 3, in the SAMRC's Ethics Committee Guidelines on Ethics for Medical Research 4th edition, the South African National Department of Health's (NDOH) 2015 Ethics in Health Research guidelines, the NDOH 2020 Guidelines for Good Clinical Practice in the conduct of clinical trials with human participants in South Africa, the principles of the Declaration of Helsinki (1964, as amended in Fortaleza Brazil, 2013), ICH Good Clinical Practice (GCP) and the laws of South Africa. No participants will be enrolled into the study until the Human Research Ethics Committee of the SAMRC and the Faculty of Health Science at the University of Cape Town has approved the

protocol, including assessment of the risks-benefits ratio. The study has been registered with the Pan African Clinical Trials Registry (PACTR) in compliance with the South African regulations (PACTR202308727853680).

6.2 Informed consent

No participant will enter the study without signing informed consent. Prior to signing consent, the investigator and/or fieldworker will provide a full and adequate oral explanation of the nature and purpose of the study, the procedures involved, and any foreseeable risks associated with participation in the study. In addition, a detailed information sheet describing the nature of the study, their anticipated role in the study, and the requirements for participation will be provided. The participants will have the opportunity to ask questions and be given adequate time (at least 24 hours) to discuss potential participation with their family before a decision is made. The participants will be made aware that they are under no obligation to participate in the study and can withdraw from the study at any point without stating a reason. In addition, the trial personnel may also withdraw a participant from the study if they violate the trial protocol. Participants withdrawing from the study will receive compensation for their time and inconvenience on a pro rata basis. In the event a participant is withdrawn from the study, permission to retain the participant's samples and data in an unlinked, anonymous manner will be obtained. Alternatively, the samples and data will be destroyed.

The possibility of future analyses of the blood, stool and urine samples and data will be discussed with the participants. New research questions may be formulated based on the results of this study of which we are at present not aware. But these questions may be important to further our understanding of the biological effects of BFP. Approval from the relevant Research Ethics Committees of the SAMRC and the Faculty of Health Sciences at the University of Cape Town will be obtained prior to any further analyses. Participants will be free to refuse future analyses of their samples and will not be penalised in any way, and the samples will be destroyed on completion of this trial.

Capacity to consent

Adults who have the capacity to make their own decisions to provide informed consent will be eligible to be included in this study. Participants will be required to display an understanding of the purpose and procedures of the study by relaying a summary of the details back to the trial coordinator and/or field worker before signing the consent form. The participant may pose questions regarding any aspect of the study.

6.3 Confidentiality and anonymity

Confidentiality will be strictly maintained. Staff and students involved in participant data collection have GCP certificates and will undergo training with regards to protection of confidential information. Participant names will be removed from all data, and each participant will receive a unique code to be used for sample and data analysis on the electronic data management system. Personal data is stored separately from the research data (questionnaire, anthropometric data, biomarkers etc.) on RedCap in a password protected and encrypted

profile. Study team members will receive password-protected permission to access the database as required. Blood samples collected from participants will be coded with the unique code by the trained phlebotomist.

Interviewers and those involved in taking the measurements will not be blinded regarding the identity of individual participants, however data processors and analysts/statisticians will not be able to identify any of the participants. All generated data will be stored in a manner that maintains participant's confidentiality. The PI will have access to personal data to ensure that individual test results can be communicated to the participants at the end of the trial. Data will be stored on the SAMRC's IT infrastructure which provides the relevant protection and backup features. Samples (blood, stool, urine) labelled with the unique participant code will be stored in -80°C freezers at the SAMRC for up to 10 years. These freezers are equipped with a temperature monitoring system and SMS alert system to ensure sample integrity over time. Permission to store data and samples for future research will be requested in the relevant consent form. The anonymity of participants will be ensured in any publication of the data.

The data collected as a part of this study will be managed by the principal investigators and shared with other researchers in future according to the policies governing data sharing and preservation by the SAMRC and UCT. A collaboration agreement including details regarding Data Transfer (DTA) and materials transfer (MTA) will be set up between SAMRC and UCT, and any other collaborating institutions to enable collaborative analysis and write up of the results of the study. All publications resulting from this project will be approved by all co-investigators prior to submission for publication. SAMRC and UCT endorse policies promoting data preservation and sharing. The principal investigators are prepared to share the dataset with the wider scientific community and will make the data available after all the results have been published, in response to appropriate requests. As there is no proprietary or patentable data generated by the study, there will be no restrictions on data sharing.

6.4 Benefits and risks to participants

6.4.1 Potential Benefits

BFP has been consumed in indigenous populations around Africa for centuries and is considered safe. The main benefits of the study will be scientific advancement of our understanding of the effect of BFP and dietary fibre on intestinal permeability and secondarily on gut microbiota and cardiometabolic risk factors in a South African population sample. While the participants are unlikely to derive any direct clinical benefit from the study, the participants will receive the results of the study as well as their individual results with respect to body composition (weight, height, waist circumference, BMI, body fat percentage), liver and kidney markers, serum lipid profile, blood pressure, and dietary analysis. If the participant has any abnormal results, he or she will be given a referral letter and directed to the appropriate health practitioner or local clinic.

6.4.2 Potential Risks and discomfort

Risks associated with consumption of Baobab fruit powder:

The BFP is commercially available as a novel food ingredient in Europe and the USA and is available on the South African market. There is a very small risk of an allergic reaction to Baobab fruit powder (Martini *et al.*, 2019). The placebo, tapioca flour, which is typically used as a food thickener in many food products, carries a very small risk. Participants will be monitored closely through weekly contacts and asked about tolerability and acceptability. Participants will be required to complete a questionnaire about their gastrointestinal symptoms biweekly. Participants will be requested to immediately inform the trial management should they experience any adverse events and will be referred to the trial doctor for medical attention at no cost to the participant. Blood samples will be taken at baseline, 2-weeks and after 45 days of the intervention to monitor liver and kidney function.

Risks associated with administering the lactulose/mannitol test:

Lactulose is an over-the-counter laxative used to treat chronic constipation and is generally well tolerated at the standard dose of 10 to 20 g per day. For the Lac/Man test a 5 g dose will be applied, which is half of the standard therapeutic dose, which is based on literature (Del Piano *et al.*, 2014; Kato *et al.*, 2017; Seethaler *et al.*, 2021). However, lactulose at a therapeutic dose may take up to 48 hours to take effect and there is a risk that participants may develop soft or loose stool, which should resolve by itself. Other side effects and allergic reactions can, on rare occasions, occur and will be monitored and participants referred to the trial doctor should any serious side effects occur.

Mannitol is a sugar alcohol which is used as a sweetener in foods. A typical dose is 4 g per serving, which can be used several times per day. For the Lac/Man test we will require a 2 g dose of mannitol, i.e. half of a normal serving, to be applied (Del Piano *et al.*, 2014; Kato *et al.*, 2017; Seethaler *et al.*, 2021). However, side effects and allergic reactions can in rare cases occur and participants will be monitored and referred to the trial doctor should any serious side effects occur.

Risks associated with blood sampling:

Only minor risks are associated with blood sampling, such as bruising or haematoma around the area, pain at the site of injection, anxiety and fainting, and local infection. All necessary precautions will be taken to minimise these risks. Participants will be seated comfortably and counselled to reduce anxiety during the blood draw. All procedures will be carried out by appropriately trained personnel using sterile techniques and the WHO guidelines on drawing blood will be followed (World Health Organization, 2010) to minimise any risks to the participants and phlebotomist. A maximum of 100 ml of blood will be drawn during the entire study, which is significantly less than a standard blood donation (450 ml).

Minimising risk:

All procedures will be supervised and carried out by an appropriately trained and experienced phlebotomist using sterile techniques to minimise any risks of infection. Prior to the study, the participants will be asked if

they have previously had any adverse reactions to BFP, lactulose and mannitol and will be excluded from the trial based on a positive response. Participants will be monitored during Lac/Man testing. A trial doctor will be on standby throughout the course of the study. Medication and equipment are readily available on an emergency trolley within the facility to stabilize a participant in the unlikely event of a medical emergency. There are AEDs and emergency alarms readily accessible on each floor of the building. Dr D'Alton will be the lead clinician at the site and is supported on-site by the research nurse, and Prof Joel Dave.

Insurance:

If a participant falls ill, suffers any side effects or if a participant is injured in any study related manner, the investigator/researcher needs to be contacted immediately and the appropriate medical care will be sourced. The participant will have the contact details of the project manager/nurse should they experience any discomfort related to the testing procedures once they leave the testing facility. The University of Cape Town has no-fault insurance cover for the event that research-related injury or harm results from participation in the trial.

6.5 Use of the results/impact

In addition to the participants receiving their individual results, the final study findings will be presented at a research feedback session. During this session, the effects (if any) of BFP on intestinal permeability, gut microbiota and cardiometabolic markers will be presented and discussed by the research team.

This is the first study to explore potential effects of regular consumption of BFP on intestinal barrier function and gut microbiota diversity in humans. Further, this study will likely advance our understanding of the relationship between intestinal permeability and cardiometabolic risk in humans. In addition, there is limited scientific evidence on the sustained effects of BFP. To date our understanding is based on observational studies or acute studies in healthy volunteers who received once-off treatments. The outcomes of the study may stimulate further research exploring the effects and mechanisms of action of BFP, including studies in populations with metabolic and other diseases.

The study will allow three emerging South African scientists to gain critical skills and expertise in state-of-the art research methods in order to complete their degrees. It is envisaged that two MSc students and a PhD student will be trained on this project.

The study will likely yield a minimum of three separate scientific peer-reviewed publications in ISI journals. All study team members will be authors on the publications:

- "Effects of Baobab fruit powder on gut and cardiometabolic health – study protocol for a randomised double-blind placebo-controlled trial"
- "Effect of Baobab fruit powder on markers of intestinal permeability and microbial diversity in South Africans with obesity: results from a randomised placebo-controlled trial"

- “Method development and validation of the urinary lactulose/mannitol ratio LC/MS in South African study participants with obesity”

7. References

Ahmed AM, Khabour OF, Yousuf A, Eweda SM, Mohammedsaeed W, Daradka HM, Hassanein SFM & Ibrahim AM (2022). The beneficial effect of Adansonia digitata products success to modulate lipid profiles and inhibit LDL oxidation in-vitro: An associational study. *Malawi Med J* **34,** 25–30.

Allali I, Abotsi RE, Tow LA, Thabane L, Zar HJ, Mulder NM & Nicol MP (2021). Human microbiota research in Africa: a systematic review reveals gaps and priorities for future research. *Microbiome* **9,** 241.

Asogwa IS, Ibrahim AN & Agbaka JI (2021). African baobab: Its role in enhancing nutrition, health, and the environment. *Trees For People* **3,** 100043.

Barengolts E, Green SJ, Chlipala GE, Layden BT, Eisenberg Y, Priyadarshini M & Dugas LR (2019). Predictors of Obesity among Gut Microbiota Biomarkers in African American Men with and without Diabetes. *Microorganisms* **7,** E320.

Brunkwall L & Orho-Melander M (2017). The gut microbiome as a target for prevention and treatment of hyperglycaemia in type 2 diabetes: from current human evidence to future possibilities. *Diabetologia* **60,** 943–951.

Camilleri M (2019). Leaky gut: mechanisms, measurement and clinical implications in humans. *Gut* **68,** 1516–1526.

Cani PD et al. (2007). Metabolic endotoxemia initiates obesity and insulin resistance. *Diabetes* **56,** 1761–1772.

Cani PD (2019). Targeting gut microbiota with a complex mix of dietary fibers improves metabolic diseases. *Kidney Int* **95,** 14–16.

Cantrell K et al. (2021). EMPress Enables Tree-Guided, Interactive, and Exploratory Analyses of Multi-omic Data Sets. *mSystems* **6,** e01216-20.

Chadare FJ, Linnemann AR, Hounhouigan JD, Nout MJR & Van Boekel M a. JS (2009). Baobab food products: a review on their composition and nutritional value. *Crit Rev Food Sci Nutr* **49,** 254–274.

Coe S & Ryan L (2016). White bread enriched with polyphenol extracts shows no effect on glycemic response or satiety, yet may increase postprandial insulin economy in healthy participants. *Nutr Res N Y N* **36,** 193–200.

Coe SA, Clegg M, Armengol M & Ryan L (2013). The polyphenol-rich baobab fruit (Adansonia digitata L.) reduces starch digestion and glycemic response in humans. *Nutr Res N Y N* **33,** 888–896.

Deehan EC et al. (2022). Elucidating the role of the gut microbiota in the physiological effects of dietary fiber. *Microbiome* **10,** 77.

Deehan EC, Yang C, Perez-Muñoz ME, Nguyen NK, Cheng CC, Triador L, Zhang Z, Bakal JA & Walter J (2020). Precision Microbiome Modulation with Discrete Dietary Fiber Structures Directs Short-Chain Fatty Acid Production. *Cell Host Microbe* **27,** 389-404.e6.

Del Piano M, Balzarini M, Carmagnola S, Pagliarulo M, Tari R, Nicola S, Deidda F & Pane M (2014). Assessment of the capability of a gelling complex made of tara gum and the exopolysaccharides produced by the microorganism

Streptococcus thermophilus ST10 to prospectively restore the gut physiological barrier: a pilot study. *J Clin Gastroenterol* **48 Suppl 1,** S56-61.

Delzenne NM, Knudsen C, Beaumont M, Rodriguez J, Neyrinck AM & Bindels LB (2019). Contribution of the gut microbiota to the regulation of host metabolism and energy balance: a focus on the gut-liver axis. *Proc Nutr Soc* **78,** 319–328.

Dias S, Adam S, Abrahams Y, Rheeder P & Pheiffer C (2021). Adiponectin DNA methylation in South African women with gestational diabetes mellitus: Effects of HIV infection. *PloS One* **16,** e0248694.

Dickie K, Micklesfield LK, Chantler S, Lambert EV & Goedecke JH (2014). Meeting physical activity guidelines is associated with reduced risk for cardiovascular disease in black South African women; a 5.5-year follow-up study. *BMC Public Health* **14,** 498.

Drucker DJ (2007). The role of gut hormones in glucose homeostasis. *J Clin Invest* **117,** 24–32.

Dugas LR, Bernabé BP, Priyadarshini M, Fei N, Park SJ, Brown L, Plange-Rhule J, Nelson D, Toh EC, Gao X, Dong Q, Sun J, Kliethermes S, Gottel N, Luke A, Gilbert JA & Layden BT (2018). Decreased microbial co-occurrence network stability and SCFA receptor level correlates with obesity in African-origin women. *Sci Rep* **8,** 17135.

Ecklu-Mensah G, Choo-Kang C, Maseng MG, Donato S, Bovet P, Viswanathan B, Bedu-Addo K, Plange-Rhule J, Oti Boateng P, Forrester TE, Williams M, Lambert EV, Rae D, Sinyanya N, Luke A, Layden BT, O'Keefe S, Gilbert JA & Dugas LR (2023). Gut microbiota and fecal short chain fatty acids differ with adiposity and country of origin: the METS-microbiome study. *Nat Commun* **14,** 5160.

Eromosele IC, Eromosele CO & Kuzhkuzha DM (1991). Evaluation of mineral elements and ascorbic acid contents in fruits of some wild plants. *Plant Foods Hum Nutr Dordr Neth* **41,** 151–154.

Evang EC, Habte T-Y, Owino WO & Krawinkel MB (2021). Can the supplementary consumption of baobab (Adansonia digitata L.) fruit pulp improve the hemoglobin levels and iron status of schoolchildren in Kenya? Findings of a randomized controlled intervention trial. *Eur J Nutr* **60,** 2617–2629.

Fan Y & Pedersen O (2021). Gut microbiota in human metabolic health and disease. *Nat Rev Microbiol* **19,** 55–71.

Fedarko MW, Martino C, Morton JT, González A, Rahman G, Marotz CA, Minich JJ, Allen EE & Knight R (2020). Visualizing 'omic feature rankings and log-ratios using Qurro. *NAR Genomics Bioinforma* **2,** lqaa023.

Foltz M, Zahradnik AC, Van den Abbeele P, Ghyselinck J & Marzorati M (2021). A Pectin-Rich, Baobab Fruit Pulp Powder Exerts Prebiotic Potential on the Human Gut Microbiome In Vitro. *Microorganisms* **9,** 1981.

Fortea M, Albert-Bayo M, Abril-Gil M, Ganda Mall J-P, Serra-Ruiz X, Henao-Paez A, Expósito E, González-Castro AM, Guagnozzi D, Lobo B, Alonso-Cotoner C & Santos J (2021). Present and Future Therapeutic Approaches to Barrier Dysfunction. *Front Nutr* **8,** 718093.

Friedewald WT, Levy RI & Fredrickson DS (1972). Estimation of the concentration of low-density lipoprotein cholesterol in plasma, without use of the preparative ultracentrifuge. *Clin Chem* **18,** 499–502.

Fuertig R, Ceci A, Camus SM, Bezard E, Luippold AH & Hengerer B (2016). LC-MS/MS-based quantification of kynurenine metabolites, tryptophan, monoamines and neopterin in plasma, cerebrospinal fluid and brain. *Bioanalysis* **8,** 1903–1917.

Garvey R, Clegg M & Coe S (2017). The acute effects of baobab fruit (Adansonia digitata) on satiety in healthy adults. *Nutr Health* **23,** 83–86.

Gervasoni J, Primiano A, Graziani C, Scaldaferri F, Gasbarrini A, Urbani A & Persichilli S (2018). Validation of UPLC-MS/MS Method for Determination of Urinary Lactulose/Mannitol. *Mol Basel Switz* **23,** E2705.

Goedecke JH, Mendham AE, Clamp L, Nono Nankam PA, Fortuin-de Smidt MC, Phiri L, Micklesfield LK, Keswell D, Woudberg NJ, Lecour S, Alhamud A, Kaba M, Lutomia FM, van Jaarsveld PJ, de Villiers A, Kahn SE, Chorell E, Hauksson J & Olsson T (2018). An Exercise Intervention to Unravel the Mechanisms Underlying Insulin Resistance in a Cohort of Black South African Women: Protocol for a Randomized Controlled Trial and Baseline Characteristics of Participants. *JMIR Res Protoc* **7,** e75.

Goedecke JH, Nguyen KA, Kufe C, Masemola M, Chikowore T, Mendham AE, Norris SA, Crowther NJ, Karpe F, Olsson T, Kengne AP & Micklesfield LK (2022). Waist circumference thresholds predicting incident dysglycaemia and type 2 diabetes in Black African men and women. *Diabetes Obes Metab* **24,** 918–927.

Gonzalez A & Knight R (2012). Advancing analytical algorithms and pipelines for billions of microbial sequences. *Curr Opin Biotechnol* **23,** 64–71.

Harris PA, Taylor R, Minor BL, Elliott V, Fernandez M, O'Neal L, McLeod L, Delacqua G, Delacqua F, Kirby J, Duda SN, & REDCap Consortium (2019). The REDCap consortium: Building an international community of software platform partners. *J Biomed Inform* **95,** 103208.

Harris PA, Taylor R, Thielke R, Payne J, Gonzalez N & Conde JG (2009). Research electronic data capture (REDCap)--a metadata-driven methodology and workflow process for providing translational research informatics support. *J Biomed Inform* **42,** 377–381.

Ismail BB, Pu Y, Fan L, Dandago MA, Guo M & Liu D (2019). Characterizing the phenolic constituents of baobab (Adansonia digitata) fruit shell by LC-MS/QTOF and their in vitro biological activities. *Sci Total Environ* **694,** 133387.

Johnson R, Dludla P, Mabhida S, Benjeddou M, Louw J & February F (2019). Pharmacogenomics of amlodipine and hydrochlorothiazide therapy and the quest for improved control of hypertension: a mini review. *Heart Fail Rev* **24,** 343–357.

Kato T, Honda Y, Kurita Y, Iwasaki A, Sato T, Kessoku T, Uchiyama S, Ogawa Y, Ohkubo H, Higurashi T, Yamanaka T, Usuda H, Wada K & Nakajima A (2017). Lubiprostone improves intestinal permeability in humans, a novel therapy for the leaky gut: A prospective randomized pilot study in healthy volunteers. *PloS One* **12,** e0175626.

Khoja KK, Aslam MF, Sharp PA & Latunde-Dada GO (2021). In vitro bioaccessibility and bioavailability of iron from fenugreek, baobab and moringa. *Food Chem* **335,** 127671.

Khoshbin K & Camilleri M (2020). Effects of dietary components on intestinal permeability in health and disease. *Am J Physiol Gastrointest Liver Physiol* **319,** G589–G608.

Komane B, Kamatou G, Mulaudzi N, Vermaak I & Fouche G (2023). Chapter 1 - Adansonia digitata. In *The South African Herbal Pharmacopoeia*, ed. Viljoen A, Sandasi M, Fouche G, Combrinck S & Vermaak I, pp. 1–39. Academic Press. Available at: https://www.sciencedirect.com/science/article/pii/B9780323997942000027 [Accessed July 26, 2023].

Kufe CN, Micklesfield LK, Masemola M, Chikowore T, Kengne AP, Karpe F, Norris SA, Crowther NJ, Olsson T & Goedecke JH (2022). Increased risk for type 2 diabetes in relation to adiposity in middle-aged Black South African men compared to women. *Eur J Endocrinol* **186,** 523–533.

Lambert EV, Goedecke JH, Bluett K, Heggie K, Claassen A, Rae DE, West S, Dugas J, Dugas L, Meltzeri S, Charlton K & Mohede I (2007). Conjugated linoleic acid versus high-oleic acid sunflower oil: effects on energy metabolism, glucose tolerance, blood lipids, appetite and body composition in regularly exercising individuals. *Br J Nutr* **97,** 1001–1011.

Lamien-Meda A, Lamien CE, Compaoré MMY, Meda RNT, Kiendrebeogo M, Zeba B, Millogo JF & Nacoulma OG (2008). Polyphenol content and antioxidant activity of fourteen wild edible fruits from Burkina Faso. *Mol Basel Switz* **13,** 581–594.

Lassenius MI, Pietiläinen KH, Kaartinen K, Pussinen PJ, Syrjänen J, Forsblom C, Pörsti I, Rissanen A, Kaprio J, Mustonen J, Groop P-H, Lehto M, & FinnDiane Study Group (2011). Bacterial endotoxin activity in human serum is associated with dyslipidemia, insulin resistance, obesity, and chronic inflammation. *Diabetes Care* **34,** 1809–1815.

Laurans L et al. (2018). Genetic deficiency of indoleamine 2,3-dioxygenase promotes gut microbiota-mediated metabolic health. *Nat Med* **24,** 1113–1120.

Liu R et al. (2017). Gut microbiome and serum metabolome alterations in obesity and after weight-loss intervention. *Nat Med* **23,** 859–868.

Machado AM, da Silva NBM, de Freitas RMP, de Freitas MBD, Chaves JBP, Oliveira LL, Martino HSD & de Cássia Gonçalves Alfenas R (2021). Effects of yacon flour associated with an energy restricted diet on intestinal permeability, fecal short chain fatty acids, oxidative stress and inflammation markers levels in adults with obesity or overweight: a randomized, double blind, placebo controlled clinical trial. *Arch Endocrinol Metab* **64,** 597–607.

Makhalanyane TP et al. (2023). African microbiomes matter. *Nat Rev Microbiol* **21,** 479–481.

Makki K, Deehan EC, Walter J & Bäckhed F (2018). The Impact of Dietary Fiber on Gut Microbiota in Host Health and Disease. *Cell Host Microbe* **23,** 705–715.

Mangwana N (2020). *The in vitro faecal evaluation of prebiotic effects of rooibos phenolic compounds on the gut microbiota of vervet monkeys (Chlorocebus pygerythrus)* (Master of Environmental Health thesis). Cape Peninsula University of Technology, Cape Town.

Martini M, Mistrello G, Amato S, Bilò MB, Agolini S, Corsi A, Tontini A & Antonicelli L (2019). Anaphylaxis to baobab fruit: the paradox of "natural healthy food." *Eur Ann Allergy Clin Immunol* **51,** 282–284.

Martino C, Shenhav L, Marotz CA, Armstrong G, McDonald D, Vázquez-Baeza Y, Morton JT, Jiang L, Dominguez-Bello MG, Swafford AD, Halperin E & Knight R (2021). Context-aware dimensionality reduction deconvolutes gut microbial community dynamics. *Nat Biotechnol* **39,** 165–168.

Matthews DR, Hosker JP, Rudenski AS, Naylor BA, Treacher DF & Turner RC (1985). Homeostasis model assessment: insulin resistance and beta-cell function from fasting plasma glucose and insulin concentrations in man. *Diabetologia* **28,** 412–419.

Mendham AE, Larsen S, George C, Adams K, Hauksson J, Olsson T, Fortuin-de Smidt MC, Nono Nankam PA, Hakim O, Goff LM, Pheiffer C & Goedecke JH (2020). Exercise training results in depot-specific adaptations to adipose tissue mitochondrial function. *Sci Rep* **10,** 3785.

Mirzababaei A, Zandkarimi R, Moradi S, Rasaei N, Amini MR, Pourreza S, Abaj F, Clark CCT, Daneshzad E & Mirzaei K (2022). The effect of Glucomannan on fasting and postprandial blood glucose in adults: a systematic review and meta-analysis of randomized controlled trials. *J Diabetes Metab Disord* **21,** 1055–1063.

Morton JT, Marotz C, Washburne A, Silverman J, Zaramela LS, Edlund A, Zengler K & Knight R (2019). Establishing microbial composition measurement standards with reference frames. *Nat Commun* **10,** 2719.

Nicol BM (1957). Ascorbic acid content of baobab fruit. *Nature* **180,** 287.

Nnam NM, Madukwe EU & Udenta EA (2011). Effect of Baobab (Adansonia digitata L.) Fruit Pulp Drink on Iron Status of School Children. *Niger J Nutr Sci* **32,** 33–36.

Olofsson LE & Bäckhed F (2022). The Metabolic Role and Therapeutic Potential of the Microbiome. *Endocr Rev* **43,** 907–926.

Pheiffer C, Dias S, Rheeder P & Adam S (2018). Decreased Expression of Circulating miR-20a-5p in South African Women with Gestational Diabetes Mellitus. *Mol Diagn Ther* **22,** 345–352.

Pheiffer C, Dias S, Rheeder P & Adam S (2019). MicroRNA Profiling in HIV-Infected South African Women with Gestational Diabetes Mellitus. *Mol Diagn Ther* **23,** 499–505.

PhytoTrade Africa (2009). *SUMMARY REPORT: Nutritional Evaluation of Baobab Dried Fruit Pulp and its Potential Health Benefits*. Available at: http://oh.q-sites.com/getattachment/Products/Baobab/PhytoTrade-Baobab-Nutritional-Summary-(1).pdf.aspx [Accessed June 21, 2022].

Rahul J, Jain MK, Singh SP, Kamal RK, Anuradha, Naz A, Gupta AK & Mrityunjay SK (2015). Adansonia digitata L. (baobab): a review of traditional information and taxonomic description. *Asian Pac J Trop Biomed* **5,** 79–84.

Ratshikombo T, Goedecke JH, Soboyisi M, Kufe C, Makura-Kankwende CBT, Masemola M, Micklesfield LK & Chikowore T (2021). Sex Differences in the Associations of Nutrient Patterns with Total and Regional Adiposity: A Study of Middle-Aged Black South African Men and Women. *Nutrients* **13,** 4558.

Ried K, Travica N, Dorairaj R & Sali A (2020). Herbal formula improves upper and lower gastrointestinal symptoms and gut health in Australian adults with digestive disorders. *Nutr Res N Y N* **76,** 37–51.

Riedel S, Pheiffer C, Johnson R, Louw J & Muller CJF (2022). Intestinal Barrier Function and Immune Homeostasis Are Missing Links in Obesity and Type 2 Diabetes Development. *Front Endocrinol* **12,** 833544.

Rita K, Bernardo MA, Silva ML, Brito J, Mesquita MF, Pintão AM & Moncada M (2022). Adansonia digitata L. (Baobab Fruit) Effect on Postprandial Glycemia in Healthy Adults: A Randomized Controlled Trial. *Nutrients* **14,** 398.

Roach LA, Meyer BJ, Fitton JH & Winberg P (2022). Improved Plasma Lipids, Anti-Inflammatory Activity, and Microbiome Shifts in Overweight Participants: Two Clinical Studies on Oral Supplementation with Algal Sulfated Polysaccharide. *Mar Drugs* **20,** 500.

Seethaler B, Basrai M, Neyrinck AM, Nazare J-A, Walter J, Delzenne NM & Bischoff SC (2021). Biomarkers for assessment of intestinal permeability in clinical practice. *Am J Physiol-Gastrointest Liver Physiol*; DOI: 10.1152/ajpgi.00113.2021.

Sharma JR, Mabhida SE, Myers B, Apalata T, Nicol E, Benjeddou M, Muller C & Johnson R (2021). Prevalence of Hypertension and Its Associated Risk Factors in a Rural Black Population of Mthatha Town, South Africa. *Int J Environ Res Public Health* **18,** 1215.

Song HE, Lee HY, Kim SJ, Back SH & Yoo HJ (2019). A Facile Profiling Method of Short Chain Fatty Acids Using Liquid Chromatography-Mass Spectrometry. *Metabolites* **9,** E173.

Stewart ML, Wilcox ML, Bell M, Buggia MA & Maki KC (2018). Type-4 Resistant Starch in Substitution for Available Carbohydrate Reduces Postprandial Glycemic Response and Hunger in Acute, Randomized, Double-Blind, Controlled Study. *Nutrients* **10,** E129.

Sze MA & Schloss PD (2016). Looking for a Signal in the Noise: Revisiting Obesity and the Microbiome. *mBio* **7,** e01018-16.

Vázquez-Baeza Y, Pirrung M, Gonzalez A & Knight R (2013). EMPeror: a tool for visualizing high-throughput microbial community data. *GigaScience* **2,** 16.

Venter P, Malemela K, Mbazima V, Mampuru LJ, Muller CJF & Riedel S (2021). An RP-LC-UV-TWIMS-HRMS and Chemometric Approach to Differentiate between Momordica balsamina Chemotypes from Three Different Geographical Locations in Limpopo Province of South Africa. *Molecules* **26,** 1896.

Venter P, Muller M, Vestner J, Stander MA, Tredoux AGJ, Pasch H & de Villiers A (2018). Comprehensive Three-Dimensional LC × LC × Ion Mobility Spectrometry Separation Combined with High-Resolution MS for the Analysis of Complex Samples. *Anal Chem* **90,** 11643–11650.

Venter P, Pasch H & de Villiers A (2019). Comprehensive analysis of tara tannins by reversed-phase and hydrophilic interaction chromatography coupled to ion mobility and high-resolution mass spectrometry. *Anal Bioanal Chem* **411,** 6329–6341.

Webel AR, Sattar A, Funderburg NT, Kinley B, Longenecker CT, Labbato D, Alam SK & McComsey GA (2017). Alcohol and dietary factors associate with gut integrity and inflammation in HIV-infected adults. *HIV Med* **18,** 402–411.

World Health Organization (2010). WHO guidelines on drawing blood: best practices in phlebotomy. Available at: https://apps.who.int/iris/handle/10665/44294 [Accessed October 15, 2022].

Xhakaza L, Abrahams-October Z, Mohammednur MM, Pearce B, Adeniyi OV, Johnson R & Benjeddou M (2020). Socio-demographic and modifiable risk factors of diabetes and hypertension among resource constrained patients from rural areas in Mdantsane Township in South Africa. *Afr Health Sci* **20,** 1344–1354.

Xu T, Wu X, Liu J, Sun J, Wang X, Fan G, Meng X, Zhang J & Zhang Y (2022). The regulatory roles of dietary fibers on host health via gut microbiota-derived short chain fatty acids. *Curr Opin Pharmacol* **62,** 36–42.

Zhang X, Zhao A, Sandhu AK, Edirisinghe I & Burton-Freeman BM (2022). Red Raspberry and Fructo-Oligosaccharide Supplementation, Metabolic Biomarkers, and the Gut Microbiota in Adults with Prediabetes: A Randomized Crossover Clinical Trial. *J Nutr* **152,** 1438–1449.

8. Annexures

8.1 Annexure – Information Sheet and informed consent forms

INFORMATION SHEET – PARTICIPATION IN A RESEARCH STUDY

Effects of Baobab fruit powder on gut and cardiometabolic health in obesity – a randomized placebo-controlled trial

Dear Sir/Madam,

We are scientists at the South African Medical Research Council (SAMRC) and the University of Cape Town (UCT) and would like to invite you to participate in a scientific research study aiming to investigate the effects of Baobab fruit powder with specific focus on the gut function and metabolism in participants with obesity.

What is the purpose of the study?

Baobab fruit powder (BFP) is a commercially available product that has been consumed in Africa for centuries and is known to contain high levels of fibre and vitamin C, among other nutrients. There is scientific evidence to suggest that dietary fibre may improve gut function and metabolism. With this study we would like to investigate the effect that regular BFP consumption has on selected markers of gut function, such as leaky gut and gut bacteria. As a secondary aim, we would also like to assess how cardiometabolic risk factors, such as blood lipids, glucose and body weight, among others, are affected by BFP consumption. In addition, we will also confirm the safety of consuming BFP.

What is Baobab fruit powder and from where is it sourced?

The BFP used in this study is in its natural form and commercially available in supermarkets in South Africa and abroad. It is harvested by rural harvesters in Limpopo and is then processed by EcoProducts in Makhado, South Africa. Baobab fruits are harvested between May and September. The Baobab fruits have a hard shell which contains the seeds that are covered in fruit powder. This powder is called the fruit pulp or fruit powder. The processing includes opening the fruit and separating the seeds from the powder. The fruit powder is further milled, sieved and then packaged. Batches are regularly tested according to standard specifications by an accredited testing laboratory, which includes testing for microbial, pesticide and heavy metal levels. The BFP is certified organic according to European Union and United States Department of Agriculture standards.

Who can participate?

Anyone (men and women) who meets the inclusion/exclusion criteria and can provide written informed consent can participate in this study.

How do we decide whether you are eligible to participate in the study?

You are eligible if you fit these criteria:

- Age between 30 and 45 years
- Body mass index (BMI) of 30 kg/m^2 or above (weight in kg divided by height in metres squared). We will calculate your BMI for you.
- Stable weight for the last 6 months (no changes in body weight of more than 5 kg or change in clothes size)

You will **not** be eligible if you fit these exclusion criteria:

- You have a history of gastrointestinal diseases (e.g. irritable bowel syndrome, coeliac disease, Crohn's disease, Ulcerative Colitis) and have had gastrointestinal surgery
- Current bacterial or viral infection
- You are a smoker (including electronic cigarettes) or chew tobacco
- You are pregnant or breastfeeding
- You use chronic medication (regular prescription medication for obesity and chronic diseases, frequent use of over-the-counter drugs such as non-steroidal anti-inflammatory drugs and laxatives)
- You have known chronic diseases that are currently being treated/untreated, e.g.:
  - autoimmune diseases,
  - hypertension,
  - diabetes,
  - dyslipidaemia,
  - heart disease
  - stroke
  - reproductive diseases,
  - thyroid diseases,
  - infectious diseases.
- You have severe allergies, known allergy to baobab, and food intolerances (e.g. sugar alcohol intolerance)
- You were prescribed oral antibiotic in the last 3 months or used fibre, prebiotics and/or probiotic supplements for the last 3 months

We will measure your weight and height to calculate your BMI.

You will be requested to bring all medication and supplements that you frequently use with you so that we can establish an accurate record.

How many people will take part in the study?

We are aiming to recruit 50 men and women who fit the above criteria and have a BMI of 30 kg/m^2 or above to this study. Of these, 25 will be randomly allocated to receive the active BFP and 25 will be randomly allocated to receive a placebo (a non-active substitute) with similar taste and energy content.

What does this study involve?

The dose of the BFP that we will test is 16 g, which is equal to 2 heaped tablespoons and will deliver approximately 8 g of dietary fibre (equivalent to the fibre content of approximately 2 medium apples). The BFP and placebo contain the same amount of energy, approximately 157 kJ (38 kcal), which is equivalent to half a

slice of bread. The placebo tastes the same as the BFP, but doesn't contain BFP, and will consist of tapioca flour with a small amount of citric acid. Neither you nor the research staff will know if you are receiving the BFP or the placebo. You will be asked to consume one sachet of the BFP or placebo per day. The easiest way to prepare the BFP or placebo is to dissolve it in up to 1 litre of water for consumption. You can also add it to smoothies, juice or cereal if this fits better into your daily routine, however, please note that BFP or placebo should not be heated. You will be required to consume one sachet of BFP or placebo every day for 45 days.

You will receive the required number of sachets for daily use every two weeks. We will contact you once a week and you will be requested to visit the research facility at the Sports Science Institute of South Africa at University of Cape Town every other week so we can monitor any symptoms, adverse events or illness. We will also measure your body weight and blood pressure at these visits. However, if you have any concerns or adverse events between the weekly monitoring, please contact our research team immediately.

Before and after the 45-days of supplementation you will undergo pre- and post-intervention testing session, which will help us to show which gut and cardiometabolic parameters may have changed in response to the consumption of the BFP or placebo. This testing session will take a maximum of 6 hours and will be carried out at Health through Physical Activity, Lifestyle and Sport Research Centre (HPALS), located at the Sports Science Institute of SA in Newlands, Cape Town.

What is expected of you for the pre- and post-intervention testing session?

Screening visits:

If you meet the inclusion criteria and are willing to participate in the trial, we will ask you to come to the research facility for a screening visit. At this point we will explain the study, procedures and what is expected of you in detail, and you will receive an information leaflet. We will also ask you detailed questions about your health status (including HIV status) to determine your eligibility. Although these questions may sound intrusive, this information is important for us the record because certain health conditions influence gut function markers that we are measuring during the study. We will also measure your weight and height and your blood pressure will be measured three times with a 2-minute interval between measures. In addition, a trained phlebotomist (personnel trained in drawing blood) will take a blood sample (~8 ml, equivalent to ~1 teaspoon). These blood samples will be used to measure your blood sugar, blood lipid (LDL cholesterol (or "bad" cholesterol) and HDL cholesterol ("good" cholesterol) and triglyceride (fat)) levels and liver and kidney markers. You will receive a referral letter from the study physician to consult a health care provider for treatment should there be abnormal results.

The testing sessions:

Once you have completed and passed the screening process, we will ask you to collect a stool sample, which should be representative of your normal bowel movements. This stool sample will be used to determine gut bacteria composition and diversity and we will determine the quantities of specific compounds that bacteria produce in the gut. We will provide you with an EasySampler kit with easy-to-follow instructions to make the sampling procedure easier and we will explain how to use this. Once you have collected the sample, we ask that you keep it in the fridge in the sealed container. As this sample needs to be stored at -80°C within 6 hours after collection, you can either bring this sample along to the testing session at the research facility or we will arrange to collect it from you.

We will ask you to avoid strenuous exercise, alcohol, non-nutritive sweeteners (e.g. in chewing gum), spicy food, caffeine (including tea), sporadic over-the-counter medication (such as headache pills or other nonsteroidal anti-inflammatory drugs) and supplement use for at least 48 hours prior to and for 24 hours following the pre- and post-intervention testing session. On these testing days, you will be asked to come to the research facility (HPALS) at the Sports Science Institute of SA in Newlands. We will ask you to not to eat or drink anything after dinner the night before, except water, so that you will be fasted for 10-12 hours. This testing session will take a maximum of 6 hours of your time, including a snack at the end.

When arriving at the laboratory, a trained phlebotomist (personnel trained in drawing blood) will take a blood sample (~45 ml, which is about 10 times less than a standard blood donation (450 ml)). These blood samples will be used to measure markers of inflammation and markers of gut permeability, as well as liver and kidney function.

You will then be asked to provide a urine sample (~30 ml). You will then undergo a lactulose/mannitol test, which is used to test the 'leakiness' of your gut. You will be asked to drink 5 g of lactulose (Dulphalac, Clicks) and 2 g of mannitol dissolved in a glass of water. We will ask you to spend the next 5 hours at the research facility to collect your urine. After the 5 hours, you will be provided with a snack and drink and then you will be free to go home. In the following 19 hours when you go home, we will ask that you collect your urine in containers that we will provide for you. These containers will be collected from you by our research assistant. The urine samples collected from you will be tested for the presence of lactulose and mannitol that will give an indication of how "leaky" your gut appears. We will compare your own results before and after the intervention.

During your visit, a research assistant will ask you detailed questions about your:

- Physical activity
- Personal health and lifestyle (use of medication and supplements, smoking and alcohol intake, nail-biting habits, stress)
- Sociodemographic information (age, employment status, living conditions, language preferance, ancestry)

Please note that some of the questions may feel intrusive, however, this information is important to collect for the study as these points can influence the results of the tests we will conduct. Going through these questionnaires will take approximately 1-2 hours, and we will complete this during the 5-hour waiting period for the lactulose/mannitol test.

In addition, we will measure your weight, height, waist circumference and hip circumference and body composition using a non-invasive bioimpedance device similar to devices used at the gym. We will also measure your blood pressure three times with a 2-minute interval between measures.

You will also be asked to complete a 3-day dietary record, which should preferably include two non-consecutive weekdays and one weekend day. Details on how to complete the dietary record will be provided by the dietician/research assistant and you will be provided with an information booklet that will provide you with further guidance. You will be requested to record all the food and drinks consumed over the 3-day period. This will include all the types/brands, preparation and cooking techniques, estimated quantities and portion sizes of all foods and drinks consumed over these 3 days.

Even though Baobab fruit powder has been consumed for centuries by humans, we would like to check your liver and kidney function before, after 2 weeks and after the 6-week intervention. This is a pre-caution for regulatory (SAHPRA) purposes. For this, we will take an additional fasted blood sample (~10 ml) after 2 weeks of consuming the intervention. This will make sure that the Baobab fruit powder or our placebo does not interfere with your normal bodily functions.

What are the benefits of participating in the study?

You will be given a free comprehensive health check. We will provide you with the results of your tests, such as information about your dietary analysis, blood sugar and lipid (fat) content, blood pressure and inflammation at end of the study when recruitment and follow-up are completed. However, should you have any abnormal results, you will be given a referral letter and directed to the appropriate health practitioner or local clinic immediately. The final study findings will be presented to you at a research feedback session. During this session general recommendations regarding diet and physical activity will also be provided by a registered dietician and exercise scientist.

Your participation in this study will contribute to further our scientific knowledge about the potential link between gut function, and cardiometabolic risk factors. These results will be published in the scientific literature. The study will provide three emerging scientists the opportunity to further their studies.

What are my rights while participating in the study?

Taking part in this study is your choice and you are not required to give a reason if you do not wish to participate. If you decide to take part in the study, you are free to withdraw at any time and without giving a reason and without prejudice. All information shared with the scientists will be confidential and anonymous. Should you not feel comfortable, you may refuse to answer any questions and you may stop during the interview and not continue. If you decide to withdraw from the study, you need to notify the study staff of your decision as soon as possible via phone, text or email using the contact details provided below. We will discuss with you what will happen to any information or samples that you have provided. If the incomplete samples and information can usefully contribute to the study, we will ask your permission to store them and use them in our analysis. Alternatively, on your request all your information and samples will be destroyed.

What are the risks when participating in this study?

Risks associated with consumption of Baobab fruit powder or Tapioca flour:

BFP is commercially available as a food across the world including Europe, USA and South Africa. There is a very small risk of an allergic reaction to Baobab fruit powder and a small risk of gastrointestinal symptoms such a diarrhea, bloating, and/or constipation, which will be monitored throughout the study.

Tapioca flour is commercially available food product that is used extensively for cooking and baking. It is popular for use in recipes for gluten-free baking. There is also a very small risk of an allergic reaction or intolerance to tapioca flour.

We will monitor you closely during the study and you will be contacted weekly to ask you if you have any adverse reactions or any other related or unrelated issues that may affect the study results. If you have any queries or

adverse reactions, we request that you contact the study staff immediately. In the unlikely event of an adverse event, we will refer you immediately to the trial doctor for care.

The BFP provided for this study is packaged in a facility that is certified according to the relevant industry standards, which is called FSSC 22000 version 5.1 – the highest standard of Food Safety System Certification. The BFP is also certified organic according to European Union and United States Department of Agriculture. Baobab fruits are harvested by rural harvesters in Limpopo and the dry pulp is milled into a powder at a facility in Makhado, South Africa. As mentioned above, the BFP is free from microbial and chemical contaminants certified by accredited testing laboratories. The Tapioca flour will be purchased from a local provider, Nature Choice.

Risks associated with administering the lactulose/mannitol test:

Lactulose is used to treat chronic constipation and is generally well tolerated at the standard dose of 10 to 20 g per day. For the Lactulose/Mannitol test, half of the standard therapeutic dose (5 g) will be applied. However, lactulose at a therapeutic dose may take up to 48 hours to take effect and there is a risk that you may develop soft or loose stool up to 48 hours after the test, which should resolve by itself. Other side effects and allergic reactions can, on rare occasions, occur and will be monitored at the research facility during the initial 5 hours. You will be referred to the trial doctor for medical treatment should any serious side effects occur. A medical doctor is part of the study team and will be on standby.

Mannitol is a sugar alcohol which is used as a sweetener in foods. A typical dose is 4 g per serving, which can be used several times per day. The Lactulose/Mannitol test will require taking half of a normal serving (2 g). However, side effects and allergic reactions can, in rare cases, occur and you will be monitored at the research facility for the initial 5 hours. You will be referred to the trial doctor for medical treatment should any serious side effects occur.

Risks associated with blood sampling:

Only minor risks are associated with blood sampling, such as bruising and local infection. All procedures will be supervised and carried out by a phlebotomist using sterile techniques to minimise any risks of infection. A maximum of 100 ml of blood will be drawn during the entire study, which is less than the volume used for a standard blood donation (450 ml).

Minimising risk:

All procedures will be supervised and carried out by appropriately trained and experienced personnel (research nurse) using sterile techniques to minimise any risks of infection. Prior to the study, you will be asked if you have previously had any adverse reactions to BFP, lactulose and mannitol and will be excluded from the trial based on a positive response. You will be monitored during Lac/Man testing. A medical doctor will be available during the testing sessions in the unlikely event that immediate medical assistance is required. There are emergency alarms and an emergency trolley available in the facility.

What will happen when the study is over?

Once sample collection for the entire study is completed, the samples will be transported and stored at the SAMRC. The collected samples and resultant data will be stored and analysed by the scientists at the SAMRC and used solely for the purpose of this study. When all participants have completed the research trial, the results of the blood tests (blood glucose, blood lipids, liver and kidney function and inflammatory markers) will be

shared with you. The other data will require a longer time to be analysed and will be shared once the analysis is complete. Data will be made public in an anonymised manner so that complete confidentiality is maintained.

What will happen with your samples (blood, urine and stool)?

Please note that a sub-sample of your stool will be sent to the University of California, San Diego, USA where Dr Jack Gilbert, Director of the Microbiome and Metagenomics Center, will conduct the bacterial DNA sequencing of the gut bacteria. The data will be sent to the SAMRC for analysis by our scientists.

We would like to ask your permission to store your samples at the SAMRC head office (Francie van Zijl Drive, Parow Valley, Bellville) for potential future biomedical research with a focus on gastrointestinal diseases, diabetes and heart disease. However, this will not include any genetic testing for the samples collected for this study. Specifically, these analyses include additional and more comprehensive markers of gut function, inflammation, cardiometabolic risk, bacterial metabolites and microbial biomarkers and analysis of the transcription of genes involved in these disease pathways. These additional analyses will provide understanding of the mechanisms underlying the effect (if any) of BFP on gut function and on cardiometabolic risk factors. As new research is conducted, the insights gained will generate new research questions which are not yet obvious to the scientists at present. These samples can provide valuable insights and answers in future, and it will therefore be valuable to store them for potential future analyses. Samples and data collected will be stored for up to 10 years and new ethical approval from both the SAMRC and UCT Research Ethics Committees will be requested prior to any future analyses.

Will there be reimbursement for transport costs, time and inconvenience?

You will be reimbursed for your time and inconvenience at a rate of R50 per hour for the pre- and post-intervention visit (R300 per visit). Travel costs (R100 per day) and refreshments will be provided. For the screening visit and the fortnightly monitoring visits, you will be reimbursed at a rate of R35 per hour plus transport costs (R100 per day). Payment for time and inconvenience will be on a pro-rata basis at the end of the study period, while transport costs will be paid at each visit.

Who will have access to the information collected?

Confidentiality will be strictly maintained. Your name will be removed from all data, and you will be assigned a number, which will be used to identify data relating to you. All records will be kept in a locked room and in a secure computer database in the research unit. Your name will not be linked to any of the data or used in any report or publication of the results.

What if something goes wrong?

If you fall ill, suffer any side effects or if you are injured in any study related manner contact the investigator/researcher and you will be referred to the trial medical doctor immediately. You will receive the contact details of the project manager/Research nurse in case you experience any discomfort related to the testing procedures once you leave the testing facility. The University of Cape Town has no-fault insurance cover for the event that research-related injury or harm results from participation in the trial. The insurer will pay all

reasonable medical expenses in accordance with the South African Good Clinical Practice Guidelines (DoH 2006), based on the Association of the British Pharmaceutical Industry Guidelines (ABPI) in the event of an injury or side effect resulting directly from participation in the study. You will not be required to prove fault on the part of the University.

The University will not be liable for any loss, injuries and/or harm that you may sustain where the loss is caused by

- The use of unauthorized medicine or substances during the study
- Any injury that results from the participant not following the protocol requirements or the instructions that the study nursing sister may give the participant.
- Any injury that arises from inadequate action or lack of action to deal adequately with a side effect or reaction to the intervention
- An injury that results from negligence on the part of the participant.

By agreeing to participate in this study, you do not give up the right to claim compensation for injury where you can prove negligence, in separate litigation. In particular, your right to pursue such a claim in a South African court in terms of South African law will be ensured. Note, however, that you will usually be requested to accept that payment made by the University under the SA GCP guideline 4.11 is in full settlement of the claim relating to the medical expenses. An injury is considered trial-related if it is caused by study activities. You must notify the study researcher of any side effects and/or injuries during the trial, whether they are research-related or other related complications.

UCT reserves the right not to provide compensation if, and to the extent that, your injury came about because you chose not to follow the instructions that were given while taking part in the study. Your right in law to claim compensation for injury where they can prove negligence is not affected. Copies of these guidelines are available on request.

How will we contact you?

Study personnel will take your contact details such as cell phone numbers for use with WhatsApp, Telegram or Signal and an email address. We will ask you which time of day you will be available for monitoring calls or contacts.

General Informed Consent Form

Effects of Baobab fruit powder on gut and cardiometabolic health in obesity – a randomised placebo-controlled trial

Declaration by the participant

By signing this form, I ……………………………………………………………………………………

	Initial those that you agree to
I agree to undergo preliminary testing to determine if I am eligible for the study	………….
I agree to take part in a research study titled: **Effects of Baobab fruit powder on gut and cardiometabolic health in obesity – a randomised placebo-controlled trial**	………….
I agree to have a blood sample taken, tested and stored for analyses (the current study)	………….
I agree to complete questionnaires about my physical activity, personal health and lifestyle, sociodemographic information and to complete a 3-day estimated dietary record.	………….
I agree to complete questionnaires about my gut health and have an additional blood sample at 2 weeks to test liver and kidney function	………..
I agree to have my body weight, height, waist and hip circumferences and body composition measured	………….
I agree to give a stool sample for testing and storage for analyses (the current study)	………….
I agree to undergo the lactulose/mannitol test and give urine samples for testing and storage for analyses (the current study)	………….
I agree that the research team may contact me in the future for a follow-up study	………….

I declare that:

- I have read or had read to me this information and consent form.
- I have had a chance to ask questions and all my questions have been adequately answered.
- I know that taking part in this study is voluntary and I have not been forced to take part. I may choose to leave the study at any time without any problems.

Furthermore, in accordance with the provisions of the **Protection of Personal Information Act 4 of 2013** (as amended), I hereby consent:

To my personal information (hereinafter 'data') being collected, processed, shared and stored in accordance with the research protocol as approved by the South African Medical Research Council's and University of Cape Town's Human Research Ethics Committee (SAMRC and UCT HREC);

To my anonymised data being shared, processed and transferred by third parties and between third parties, and where relevant beyond the jurisdictional borders of South Africa;

To all findings and results flowing from my anonymised data being broadly shared and published on the conclusion of the research.

Signed at:	____________	on (date)	____________
Name of participant	____________	Signature of participant	____________
Name of witness	____________	Signature of witness	____________
	____________		____________

Declaration by investigator (or person designated)

I (name) .... declare that I have explained the information in this document to ……………………………………………… I have encouraged him/her to ask questions and took adequate time to answer them. I am satisfied that he/she adequately understands all aspects of the research, as discussed above.

Signed at:	____________	on (date)	____________
Name of consent taker:	____________	Signature of consent taker:	____________

Signed at:	____________	on (date)	____________
Name of investigator	____________	Signature of investigator	____________

Time of informed consent:

CONSENT FOR STORAGE AND FUTURE USE OF UNUSED SAMPLES:

Additional consent to:

Effects of Baobab fruit powder on gut and cardiometabolic health in obesity – a randomised placebo-controlled trial

Dear Sir/Madam,

We are scientists at the South African Medical Research Council (SAMRC) and University of Cape Town (UCT) and would like to invite you to participate in a scientific research study aiming to investigate the effects of Baobab fruit powder with specific focus on gut function and metabolism in participants with obesity.

<u>Background Information:</u>

We are seeking permission to store your unused blood, urine and stool samples for possible future research, either in our own biomedical research or collaborators' research studies in diseases such as gastrointestinal disease, diabetes and heart disease. However, this will not include any genetic testing for the samples collected for this study. These analyses will be performed to explore the mechanisms of action (if any) of BFP and include additional and more comprehensive markers of gut permeability and gut bacteria, inflammation, cardiometabolic health, bacterial metabolites and microbial biomarkers and analysis of the transcription of genes involved in these disease pathways. Permission to use these samples is in addition to the use of your samples for the current study. As new research is conducted, the insights gained will generate new research questions which are not yet obvious to the scientists at present. These samples can provide valuable insights and answers in future, and it will therefore be valuable to store them for potential future analyses. New ethical approval from both the SAMRC and UCT Research Ethics Committees will be requested prior to any future analyses. Please be aware that the samples will not be sold for profit.

You will receive a unique code that will be used for sample and data analysis to ensure your confidentiality.

You may also refuse to allow future analyses of samples without being penalised, and your results relating to the current study will not be compromised in any way. If you refuse to allow future analyses of samples, your samples will be destroyed on completion of this trial. Furthermore, you may withdraw permission to use your samples at any time.

All information collected during the study will be treated with the strictest confidentiality and will only be used for scientific research purposes. All samples will be kept in a freezer in a secure facility with access limited to research personnel; all records will be kept in a locked room and in a secure computer database in the research unit. Your name will not be used in any publication of the results. For data verification and quality control purposes regulatory authorities and/or members of the SAMRC's or University of Cape Town Faculty of Health Sciences Human Research Ethics Committee may be allowed access to your personal data under conditions of strict confidentiality.

<u>Certificate of Consent:</u>

If any of the **BLOOD** that I have provided for this research project is unused or leftover when the project is completed (Tick **one** choice from each of the following boxes)

☐ I wish my **blood** sample to be destroyed immediately.

☐ I give my permission for my **blood** sample to be stored for up to 10 years and used in future biomedical research, which has been properly approved

If any of the **Urine** that I have provided for this research project is unused or leftover when the project is completed (Tick **one** choice from each of the following boxes)

☐ I wish my **urine** sample to be destroyed immediately.

☐ I give my permission for my **urine** sample to be stored for up to 10 years and used in future biomedical research, which has been properly approved

If any of the **STOOL sample** that I have provided for this research project is unused or leftover when the project is completed (Tick **one** choice from each of the following boxes)

☐ I wish my **stool** sample to be destroyed immediately.

☐ I give my permission for my **stool** sample to be stored for up to 10 years and used in future biomedical research, which has been properly approved

I have read the information, or it has been read to me. I have had the opportunity to ask questions about it and my questions have been answered to my satisfaction. I consent voluntarily and understand that I have the right to withdraw my consent without this affecting the current research study or my medical care.

Print Name of Participant __________________________

Signature of Participant ____________________________

Date ____________________________

8.2 Annexure - Steering Committee Charter v1 October 2023

Baobab Study - Steering Committee Charter

Study information

Study Title	Effects of Baobab fruit powder on gut and cardiometabolic health in obesity – a randomised placebo-controlled trial
Protocol	Baobab Study
Protocol number	SAHPRA 20230911

Steering Committee Membership

Membership consists of four experts who are independent to the trial and have no financial, scientific, or other conflict of interest with the trial, and two members of the study team involved in day to day management at the site. The SteerCo members collectively have experience in the conduct and monitoring of randomized clinical trials.

Introduction

This Charter applies to the Steering Committee (SteerCo) for the study titled "Effects of Baobab fruit powder on gut and cardiometabolic health in obesity – a randomised placebo-controlled trial", which will be conducted at the Health through Physical Activity, Lifestyle and Sport Research Centre (HPALS), Division of Physiological Sciences, Department of Human Biology, University of Cape Town (UCT), based at the Sports Science Institute of SA (SSISA) in Newlands. The purpose of this charter is to define the roles and responsibilities of the Steering Committee and outlines communication strategies and monitoring guidelines to be implemented by the steering committee.

Responsibilities of the Steering Committee

The SteerCo is responsible to provide oversight over the conduct of the trial and for safeguarding the interests of study participants. The Steering Committee will be responsible for (i) assessing the safety aspects of study procedures (e.g. review of the research protocol, informed consent documents and plans for data safety and monitoring), (ii) for monitoring the overall conduct of the study (periodic assessment of participant recruitment and retention) and (iii) report on safety and progress of the trial.

The SteerCo includes four independent experts and two study team members and is required to provide recommendations about starting, continuing, and stopping the study. The steering committee may request additional information and is asked to make recommendations about:

- Benefit/risk ratio of procedures and participant burden
- Selection, recruitment, and retention of participants
- Adherence to protocol requirements
- Completeness, quality, and analysis of measurements
- Amendments to the study protocol and consent forms
- Participant safety
- Notification of and referral for abnormal findings
- Budget
- Timeline

Organization, Interactions and Reporting

Communication with SteerCo members will be primarily through the study coordinator. It is expected that other study investigators will not communicate with SteerCo members about the study directly, except for the PI when making presentations or responding to questions at SteerCo meetings or during conference calls. The study coordinator will also take minutes of the meetings. Members of the trial management committee will be responsible for preparing a report to the SteerCo (including recruitment data, adverse events, protocol violations, laboratory data and clinical data where appropriate, see section 7) to be submitted at least one week prior to the scheduled meeting. The statistician will use the randomisation schedule to generate data tables for participant safety aspects for this report that will keep blinding intact. Unblinded reports will only be made available should there be a compelling need to make an informed decision based on the SteerCo recommendation to stop or suspend the study. A report by the SteerCo containing recommendations for continuation or modifications to the study will be forwarded to the principal investigator and study coordinator within one week of the meeting. The principal investigator and study coordinator will be responsible for distributing the formal SteerCo recommendation report to all co-investigators and to ensure that copies are submitted to all appropriate institutional and regulatory entities.

Scheduling, Timing, and Organization of Meetings

The meetings will be held online as tele- or videoconferences. The first meeting will take place before initiation of the trial and agenda points will include: (i) discussion and review of this Charter, (ii) approve commencement of the trial and (iii) to establish and/or confirm guidelines for monitoring the trial.

Meetings will be held during recruitment at selected milestones, e.g. when a third (16) of participants have been enrolled, when half of the participants are enrolled (25) and when interim safety results are available for 16, 25 and 50 participants. An emergency meeting may be called at any time in case questions regarding participant safety are encountered.

The SteerCo will meet at the end of the study period when follow up is completed for all participants. Meetings may be attended, when appropriate, by the investigators, study statistician and data managers.

Formal minutes: The study coordinator is responsible for the accuracy and transmission of the formal SteerCo minutes. These minutes are prepared to summarize the key points of the discussion and debate, requests for additional information, response of the investigators to previous recommendations, and the recommendations from the current meeting.

The SteerCo will review adverse event data, other safety data, enrolment data, interim analyses regarding safety aspects and quality and completeness of study data at each meeting to ensure proper trial conduct (compare section 7).

It is expected that all SteerCo members will attend every meeting. However, it is recognized that this may not always be possible. Quorum for voting is considered to be half the number of standing members plus one.

Stopping rules:

The SteerCo will recommend stopping the study should there be:

- 10 or more serious adverse events judged by the study physician to be related to the study investigational product

The following are recommendations for individual participants. Participation in the trial will be stopped for individual participants should there be:

- Any renal dysfunction detected
- Study-related allergic reaction
- Liver function tests results > 2-fold above the upper normal limit

- Gastro-intestinal symptoms with no other obvious cause that warrant discontinuation of the investigational product and further participation in the study (abdominal pain, nausea or diarrhoea more than 3 times per week in 2 consecutive weeks and significantly more frequent than at baseline.

Discussion of Confidential Material

Reports, discussions, minutes and any other proceedings of the SteerCo are confidential. Members of the SteerCo and other ad hoc participants are expected to maintain confidentiality. Should a member leave the SteerCo during the study, they will be expected to maintain confidentiality regarding the participation in the SteerCo and a suitable replacement will be appointed.

List of data tables to be reviewed at the meetings:

- Summary of screening (enrolment status, failures)
- Summary of adherence and drop-out
- Adverse events
- Summary of screening and baseline results per study arm (Results from blood tests, gastro-intestinal symptom questionnaires, Bristol stool chart)
- Summary of interim results (Results from blood tests, gastro-intestinal symptom questionnaires, Bristol stool chart)
- Budget report

Amendments and review of the charter

The charter will be reviewed at the initial meeting before recruitment commences. Reviews of the charter can be added to the agenda of meeting ad hoc when required and will be included in the meetings when 25 participants have been recruited and when interim results for 25 participants are available.
